# Isorhamnetin Alleviates Renal Fibrosis by Inducing Endogenous Hydrogen Sulfide and Regulating Thiol-Based Redox State in Obstructed Kidneys

**DOI:** 10.3390/biom14101233

**Published:** 2024-09-29

**Authors:** Zhen Zhang, Haiyan Zhang, Jianyu Shi, Zheng Wang, Yanni Liang, Jingao Yu, Hongbo Wang, Zhongxing Song, Zhishu Tang, Dongbo Zhang, Jian Yao

**Affiliations:** 1 Shaanxi Collaborative Innovation Center of Chinese Medicinal Resources Industrialization, Shaanxi University of Chinese Medicine, Xianyang 712046, China; zhen@sntcm.edu.cn (Z.Z.); 223120012130@email.sntcm.edu.cn (H.Z.); 223040022984@email.sntcm.edu.cn (J.S.); 1501001@sntcm.edu.cn (Z.W.); 1501009@sntcm.edu.cn (Y.L.); 1501028@sntcm.edu.cn (J.Y.); 1612112@sntcm.edu.cn (Z.S.); 1211010@sntcm.edu.cn (Z.T.); 2 Department of the Thyroid and Breast Surgery, Xianyang Central Hospital, Xianyang 712000, China; whb080101@163.com; 3 Beijing University of Chinese Medicine, Beijing 102488, China; 4 Division of Molecular Signaling, Department of the Advanced Biomedical Research, Interdisciplinary Graduate School of Medicine, University of Yamanashi, Chuo 409-3898, Japan

**Keywords:** isorhamnetin, renal fibrosis, hydrogen sulfide, sulfhydration, keap1

## Abstract

Isorhamnetin (ISO) is an active flavonoid compound mainly isolated from the fruits of *Hippophae rhamnoides* L. and the leaves of *Ginkgo biloba* L. Previous studies have revealed the antifibrotic action of ISO in the liver and lungs, although its potential protective effects against renal fibrosis and the underlying mechanisms are still poorly understood. Given that many actions of ISO could be similarly attained by hydrogen sulfide (H_2_S), we speculated that ISO may work through the induction of endogenous H_2_S. To test the hypothesis, we established the unilateral ureteral obstruction (UUO) renal fibrosis rat model and transforming growth factor-β1(TGF-β1)-induced fibrosis in cultured renal tubular cells. ISO treatment inhibited epithelial–mesenchymal transition (EMT) formation, decreased extracellular matrix (ECM) deposition, and relieved renal fibrosis. Further analysis revealed that ISO stimulated the expression of the H_2_S-synthesizing enzyme cystathionine lyase (CSE) and cystathionine beta-synthase (CBS), and promoted H_2_S production in vivo and in vitro. The elevated H_2_S attenuated oxidative stress and elevated the thiol level. It induced Keap1 sulfhydration, disrupted Keap1-Nrf2 interaction, and promoted the entry of Nrf2 into the nucleus. Finally, we found that circulating H_2_S mainly derived from the liver, and not the kidney. Collectively, our study revealed that ISO alleviated renal fibrosis by inducing endogenous H_2_S and regulating Keap1-Nrf2 interaction through sulfhydration of Keap1. Endogenous H_2_S could be an important mediator underlying the pharmacological actions of ISO. Due to the multifunctional properties of H_2_S, the H_2_S-inducing nature of ISO could be exploited to treat various diseases.

## 1. Introduction

Tubulointerstitial fibrosis is the final common pathway to chronic kidney disease (CKD). Pathologically, renal fibrosis is associated with the formation of epithelial–mesenchymal transition (EMT) and the excessive deposition of the extracellular matrix (ECM), inducing an ongoing loss of normal tissue structure in a scarring process that leads to end-stage renal disease [1]. However, the mechanisms involved have yet to be fully understood, and the available therapeutics are limited and often come with serious side effects. Therefore, it is crucial to clarify the relevant mechanisms and find more effective and advanced antifibrotic medications.

Hydrogen sulfide (H_2_S) is an important gaseous signaling molecule that is naturally synthesized from cysteine or homocysteine via the trans-sulfuration pathway by enzymes such as cystathionine lyase (CSE) and cystathionine beta-synthase (CBS) [2]. H_2_S has multiple biological functions, including vasodilation [3], anti-inflammatory [4], neuroprotection [5], and antioxidation actions [2]. Several recent studies have shown that H_2_S exhibits antifibrotic effects in several different animal models, such as obstructed nephropathy [6], 5/6 nephrectomy [7], and diabetic nephropathy [8]. The antifibrotic effects of H_2_S are primarily due to its anti-inflammatory, antioxidative, and transforming growth factor-β1(TGF-β1)-signaling blocking effects. Thus, regulating endogenous H_2_S could be a potential way to alleviate the progression of kidney fibrosis [9,10].

Isorhamnetin (ISO) is one of the most important active flavonoids mainly isolated from the fruits of *Hippophae rhamnoides* L. and the leaves of *Ginkgo biloba* L. It is a derivative of 2-phenylchromogenone with a wide range of pharmacological actions on vascular systems, including vasodilation, anti-inflammatory, neuroprotection, and antioxidation actions. It also exhibits pharmacodynamics against several diseases, including hypotension, atherosclerosis, myocardial ischemia, and thrombosis [11,12]. Currently, it has been revealed that ISO attenuates liver fibrosis by inhibiting TGF-β/Smad signaling and relieving oxidative stress [13]. ISO protects against bleomycin-induced pulmonary fibrosis by inhibiting endoplasmic reticulum stress and EMT [14]. Therefore, we hypothesized that ISO would also attenuate renal fibrosis. Additionally, both ISO and H_2_S exhibit similar pharmacological actions, such as vasodilation, anti-inflammation, and antioxidation. Both compounds have been proven effective in attenuating fibrosis based on common pathologies, such as TGF-β signaling, oxidation, and inflammation [2,3,4,5,9,10,11,12,13,14]. We further hypothesized that the effects of ISO could be mediated by H_2_S. The purpose of this study was to test these hypotheses.

Here, we present our results that ISO attenuated renal fibrosis in vivo and in vitro through mechanisms involving induction of endogenous H_2_S and H_2_S-mediated antioxidant actions. The property of ISO could be exploited to treat a wide range of diseases.

## 2. Materials and Methods

### 2.1. Materials

Isorhamnetin was obtained from the National Institutes for Food and Drug Control (Beijing, China). Recombinant human TGF-β1 was obtained from InvivoGen (San Diego, CA, USA). Antibodies against the alpha-smooth muscle actin (α-SMA), E-cadherin, fibronectin, and TGF-β were purchased from Cell Signaling Technology (Beverly, MA, USA). Collagen-I and Nrf2 antibodies were obtained from Abcam (Cambridge, UK). H_2_S assay kit was purchased from Nanjing Jincheng Bioengineering Institute (Nanjing, China), CSE antibody was obtained from Gene Tex (Irvine, SC, USA), and CBS antibody was purchased from Santa Cruz Biotechnology (Santa Cruz, CA, USA). GAPDH was obtained from Proteintech Group (Wuhan, China). All other chemicals were from Beyotime Biotechnology (Shanghai, China).

### 2.2. Animals and Experimental Protocols

Male Sprague–Dawley rats (200 ± 10 g) were purchased from the Chengdou dossy experiment Animals CO., LTD (Chengdou, China). Unilateral ureteral obstruction (UUO) operation was performed as described previously [15]. Rats were randomly divided into five groups: sham, UUO, UUO with enalapril (10 mg/kg/day), and UUO with ISO (10 mg/kg/day), ISO was given intraperitoneally once daily 3 days before surgery and continued for 14 days after operation. The renin–angiotensin system inhibitor enalapril has been proven to have an anti-renal fibrosis effect, and it was commonly administered intragastrically as a positive control in renal fibrosis research [6]. Sham and UUO rats received saline treatment. All rats were sacrificed 14 days post-UUO. The kidney tissues were harvested and plasma was collected accordingly. The whole kidney was coronally cut in the middle. One half was used for histological studies and the other half for Western blotting analysis. The plasma was used to detect the H_2_S levels. The experimental protocols were approved by the Institutional Animal Care and Use Committee of Shaannxi University of Chinese Medicine.

### 2.3. Cell Culture

Normal rat kidney epithelial (NRK-52E) and HepG2 cells were purchased from the National Collection of Authenticated Cell Culture (Shanghai, China) and maintained in Dulbecco’s modified Eagle’s medium (4.5 g/L glucose) supplemented with 5% fetal bovine serum (Kibbutz Beit Haemek, Israel). For experiments, cells were cultured with high glucose DMEM containing 1% FBS in the presence or absence of various stimuli.

### 2.4. Immunohistochemical Staining and Histological Analysis

The left kidney tissue was fixed in 4% paraformaldehyde buffer for 24 h, embedded in paraffin, sectioned, and subjected to Masson staining with Masson trichrome reagents. Renal tubule dilation was observed, and randomly selected fields from 4 animals were observed under a light microscope. Semiquantitative analysis of interstitial extracellular matrix deposition was performed using NIH Image J software (http://rsb.info.nih.gov/ij, accessed on 12 January 2023) and the blue-positive staining of collagen fibers was evaluated.

For immunohistochemical staining, renal sections were incubated with a-SMA (1:1000) and fibronectin (1:500) primary antibodies overnight at 4 °C and then incubated with the secondary antibodies at room temperature for 1 h. Diaminobenzidine (DAB) was used to visualize positive proteins, which appeared brownish yellow. Hematoxylin was utilized for nuclear blue staining. Images of α-SMA and fibronectin staining were captured and analyzed using NIH Image J software.

### 2.5. Endogenous H_2_S Measurement

Plasma H_2_S levels were detected with the methylene blue method by using an endogenous H_2_S assay kit. Briefly, plasma was collected and separated without chyle or hemolysis on the same day. For each sample, 300 µL of plasma was used, while the blank control consisted of 300 µL of distilled water. The H_2_S assay kit instructions were then followed for use. The absorbance of the final solution was measured at the wavelength of 670 nm with a spectrophotometer.

Cell and tissue H_2_S production was measured with the same H_2_S assay kit as described above. Briefly, cells were seeded in a 12-well plate at a concentration of 3 × 10^5^/mL for 24 h. After cell stimulation, the cellular lysate was extracted with 1 mL extracting solution per well and sonicated in an ice bath. Each rat’s left kidney was weighed, and 0.1 g of it was added to 1 mL of extracting solution and homogenized using a homogenizer. Both cells and tissue extracts were centrifuged at 4 °C and 12,000 rpm for 10 min. The supernatant was harvested on ice and subjected to detection of H_2_S production using the H_2_S assay kit. The OD was determined at 670 nm with a spectrophotometer. The H_2_S concentration was calculated following the instructions.

Cells were exposed to H_2_S detection probe Hsip-1 DA (5 µM) with or without ISO for 30 min. After washing the cells, the fluorescent images of cells were captured using a fluorescence microscope (Olympus, Tokyo, Japan). The cellular fluorescent intensity was detected using a microplate reader (Thermo, Multiskan).

### 2.6. Cell Proliferation Assay

Cell proliferation was assessed using cell counting kit-8 (CCK-8). For the CCK-8 assay, cells (2.0 × 10^5^/mL) were seeded into 96-well culture plates with 1% FBS medium for 24 h, and then cells were pretreated with different concentrations of ISO (2.5–50 µM) for 30 min, followed by incubation with or without 10% FBS for 48 h. WST-8 reagent (10 µL) was added to each well. When the slightly yellow WST-8 was converted to an orange–yellow formazan dye, OD was measured by a spectrometer at the wavelength of 450 nm.

### 2.7. Protein Oxidation Assay

Protein oxidation was evaluated using the OxyBlot Protein Oxidation Detection Kit (EMD Millipore, Billerica, MA, USA). Briefly, the cellular protein was extracted using a RIPA lysis buffer (Beyotime Biotechnology, Shanghai, China) containing 50 mM DTT. The samples (5 µL) were mixed with 12% SDS (5 µL) for denaturalization and 1× DNPH (2, 4-dinitrophenylhydrazine) solution (10 µL) for derivatization. The reaction proceeded for 15 min at room temperature, and 7.5 µL neutralization solution was added for neutralization. The negative control was prepared using the 1× derivatization control solution instead of the 1× DNPH solution. The treated sample and the negative control were subjected to Western blot analysis.

### 2.8. Detection of Reactive Oxygen Species (ROS) Production

The generation of ROS and superoxide anion (O_2_^−^) was detected by a ROS/Superoxide detection kit from Enzo (Lausen, Switzerland, ENZ-51010) following the manufacturer’s instructions [16]. The ROS detection dye (green probe) reacts directly with a wide range of reactive species, such as hydrogen peroxide (H_2_O_2_), peroxynitrite (ONOO^−^), hydroxyl radicals (HO), nitric oxide (NO), and peroxy radical (ROO), yielding a green fluorescent product indicative of cellular production of different ROS/RNS types. Briefly, cells were seeded in 96-well plates (2.5 × 10^5^/mL) and cultured for 24 h. After preincubation of the cells with ISO (25 μM) for 30 min, TGF-β1(2 ng/mL) was added for 24 h, which was followed by incubation with oxidative stress detection reagent (green) for an additional 30 min. After washing the cells with 1× wash buffer, the immunofluorescent images were visualized and captured by an immunofluorescent microscope. ROS (Ex/Em: 490/525 nm) was measured using a microplate reader (Thermo, Multiskan).

### 2.9. Coimmunoprecipitation (Co-IP)

NRK-52E cells were lysed with a specific lysis buffer for immunoprecipitation assays. An equal amount of protein was allowed to incubate with anti-Keap1 antibody (1:50) at 4 °C for 10 min, followed by the addition of Protein A/G magnetic beads and incubation overnight at 4 °C with gentle mixing. The precipitated proteins in the beads were washed four times, eluted in the loading buffer via boiling for 5 min, and subjected to immunoblotting analysis.

### 2.10. Immunofluorescence Staining

Following the stimulation, the cells were washed with PBS and then fixed in 4% paraformaldehyde for 15 min. Subsequently, the cells were permeabilized with 0.3% Triton X-100 for 30 min. After blocking the cells with 3% BSA at room temperature for 1 h, the cells were incubated with primary antibody overnight at 4 °C. The following day, the cells were washed and incubated with Corallite 488-conjugated goat anti-rabbit IgG (Proteintech, SA00013-2, 1:1000) or Alexa Fluor 488-conjugated goat anti-mouse IgG (Beyotime, A0428, 1:250) for 1 h at room temperature. The nuclei were stained with DAPI. The image was captured and photographed under an Olympus IX71 microscope.

### 2.11. Maleimide-Labeling Assay

Cell lysates were extracted and allowed to react with 2 µM Alexa Fluor 680-conjugated C2 maleimide (red) at 4 °C for 2 h with gentle mixing. Afterward, the samples were separated using 10% SDS-PAGE and then transferred onto PVDF membranes. The fluorescent band was detected using an Odyssey^®^ CLx Infrared Imaging System (LI-COR, Lincoln, NE, USA). Ponceau S staining was conducted to verify equal protein loading.

### 2.12. Maleimide Assay

To examine the sulfhydration of Keap1, we employed an assay using Alexa Fluor 680-conjugated C2 maleimide [17]. The assay protocol involves the labeling of sulfhydryl (-SH) and sulfhydrated (-SSH) groups of Cys in keap1 with red fluorescent maleimide to form (-S-Mal) and (-S-S-Mal), and the disulfide bonds in (-S-S-Mal) are cleaved using DTT. Consequently, the reduction in fluorescence intensity (-S-Mal) indicates the level of sulfhydration. Briefly, cell lysates were extracted with a specific lysis buffer, assayed for protein concentration, and an equal amount of protein was subjected to immunoprecipitation as described above. Afterward, beads were washed with the same lysis buffer and allowed to incubate with 2 µM Alexa Fluor 680-conjugated C2 maleimide (red) for 2 h at 4 °C with gentle mixing. After washing off the unlabeled maleimide, the precipitated keap1 was redissolved in lysis buffer with or without 1 mM DTT for 1 h at 4 °C. The beads were washed, resuspended in 60 µL electrophoresis sample buffer, and boiled for 5 min to elute the protein. The supernatants were harvested and suspended in SDS-PAGE buffer for separation. After subsequently transferring to a PVDF membrane, the fluorescent signal was captured with an Odyssey^®^ CLx Infrared Imaging System (LI-COR, Lincoln, NE, USA). β-actin was used to ensure equal protein loading.

### 2.13. Western Blot Analysis

Total kidney tissue and cellular protein were extracted using RIPA lysis buffer (Beyotime Biotechnology, Shanghai, China) containing a freshly added proteinase inhibitor cocktail. The kidney tissue homogenate and cell lysates were centrifuged at 13,200× *g* for 10 min at 4 °C. The supernatant was harvested and determined for protein assay using the Micro BCA Protein Assay Kit (Beyotime Biotechnology, Shanghai, China).

Western blot was performed by the Enhanced Chemiluminescence (ECL) system. Briefly, extracted cellular proteins were separated by SDS-PAGE and transferred to a PVDF membrane. After blocking with 3% BSA or 5% milk in PBS/T, the membranes were probed directly with the primary antibodies at 4 °C overnight and then the HRP-labelled secondary antibodies at room temperature for 1 h. After washing, the bands were visualized by the chemiluminescent HRP substrate (Millipore, Bedford, MA, USA). The chemiluminescent signal was captured with a ChemiDoc XRS+ Gel Imaging System (Bio-Rad, Hercules, CA, USA) and quantified with the NIH Image J software. β-actin and GAPDH were probed to confirm the loading of equal amounts of proteins. Original western blots can be found at Figures S1–S13.

### 2.14. Statistical Analysis

All data are presented as mean ± SE. Differences between two groups were tested by Student’s *t*-test. When more than two groups were compared, one-way ANOVA with Dunnett’s test was used. *p* values less than 0.05 were considered significant.

## 3. Results

### 3.1. ISO Ameliorates UUO-Induced Profibrotic Changes

**Figure 1:**
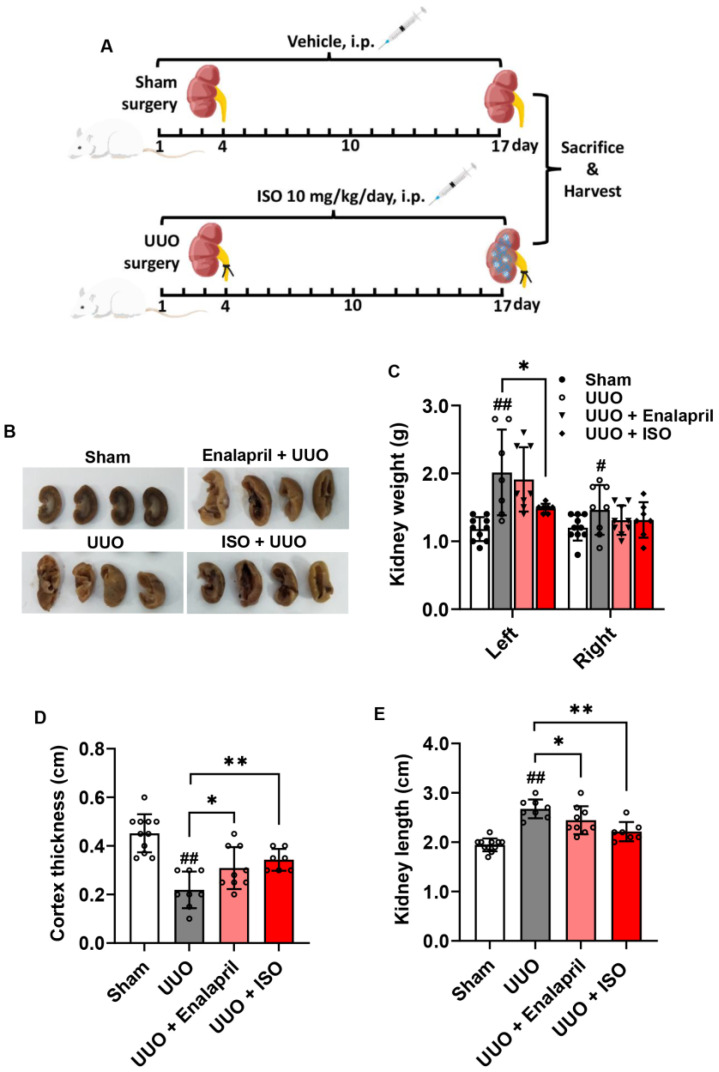
ISO treatment improves UUO-induced renal morphological changes. (**A**) The experimental outline is schematically represented. (**B**) The general appearance of four representative left kidneys from rats subjected to various treatments. Sham and UUO rats treated with vehicle (saline), ISO (10 mg/kg/day) was administered intraperitoneally, and enalapril (10 mg/kg/day) was administered intragastrically once daily before and continued for 14 days after operation. (**C**) Weights of the left and right kidney counterparts of the same rat in different groups. (**D**) Renal cortex thickness in the middle of the left kidney in the coronal section. (**E**) Lengths of left kidneys. Data shown are mean ± SE (*n* = 7–11), # *p* < 0.05, ## *p* < 0.01 versus sham group; * *p* < 0.05, ** *p* < 0.01 versus UUO group.

To determine whether ISO supplementation affects renal fibrosis, we established rat UUO models and treated them with or without ISO. The outline of the experiments is schematically depicted in Figure 1A. Rats with UUO showed increases in renal length and weight, along with a decrease in renal cortical thickness compared to sham group rats. However, treatment with ISO at 10 mg/kg/day reversed these changes, reduced renal length and weight, and increased renal cortical thickness in UUO group rats. The right kidney weight in the UUO group increased, but there were no differences in the other groups compared to the sham group (Figure 1B–E). This result indicates that ISO treatment ameliorated the morphology of the obstructed kidneys.

**Figure 2:**
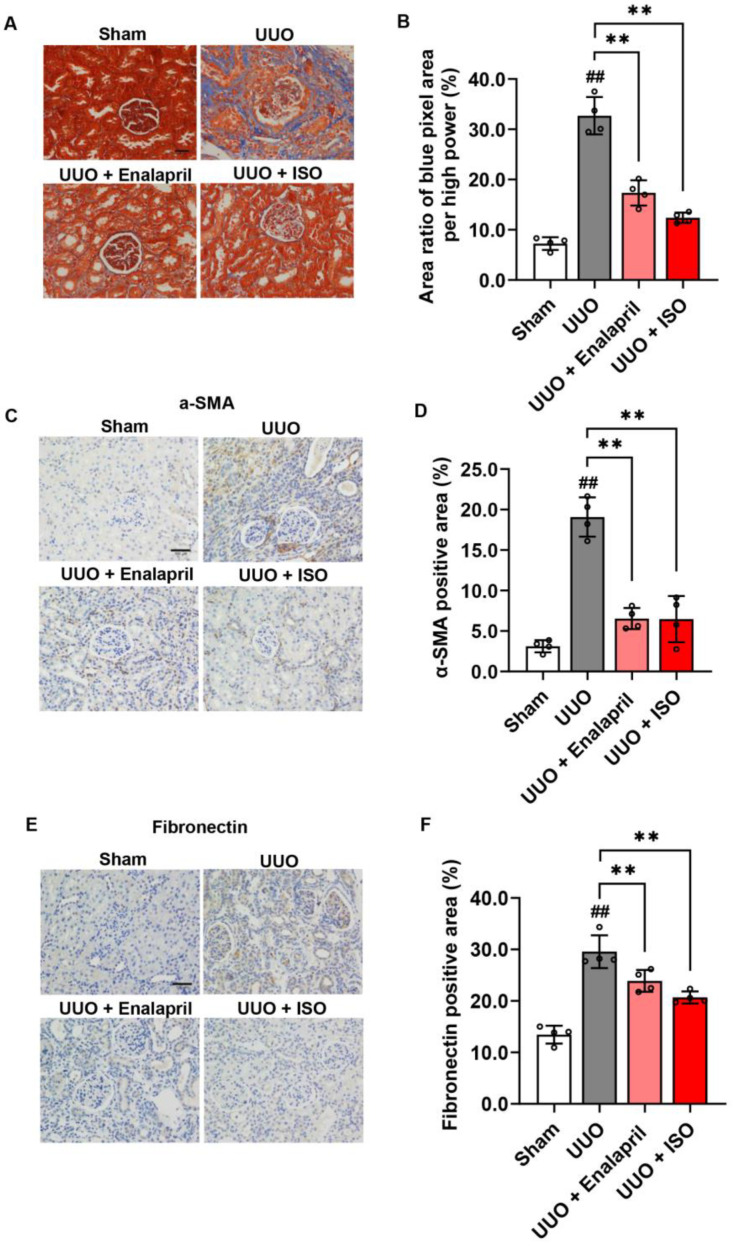
ISO attenuates the deposition of collagen fibrils and α-SMA in the obstructed kidneys 14 days after UUO operation. Representative pictures of (**A**) Masson trichrome staining and (**B**) semiquantitative analysis of the proportion of the blue color area relative to the entire field area in all groups are shown. Representative immunohistochemistry images demonstrating the expression of (**C**) α-SMA and (**E**) fibronectin, along with semiquantitative analyses of these two proteins, are presented in (**D**,**F**). Scale bar = 100 μm. Data shown are mean ± SE (*n* = 4), ## *p* < 0.01 versus sham group; ** *p* < 0.01 versus UUO group.

**Figure 3:**
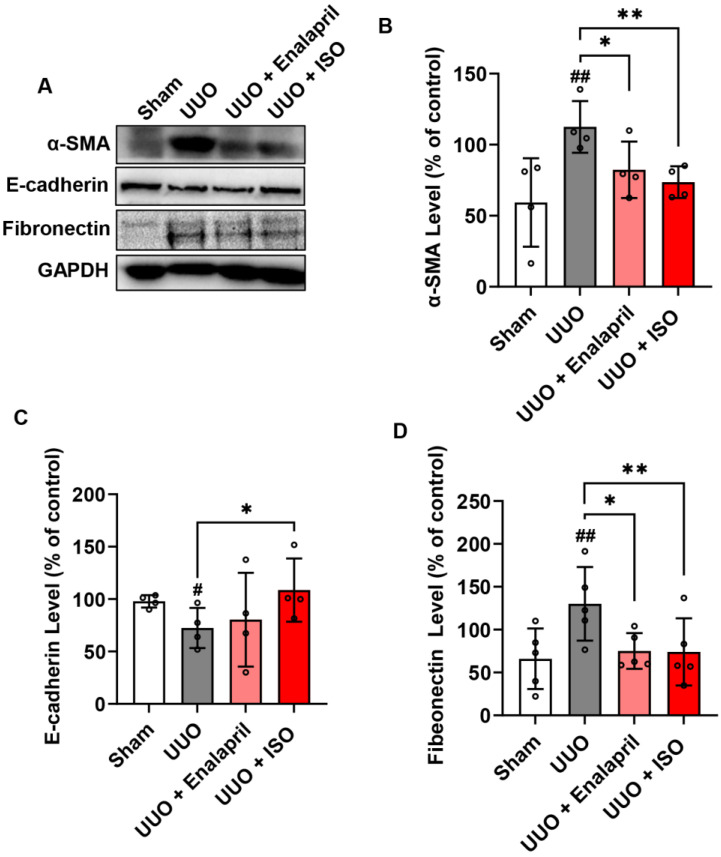
ISO inhibits EMT formation and ECM deposition in the obstructed kidneys 14 days after UUO operation. Kidney tissue lysates were assayed for the level of (**A**) α-SMA, E-cadherin, and fibronectin by Western blot, and the quantitative relative densitometric analyses of these proteins in the various groups are presented (**B**–**D**). Data shown are mean ± SE (*n* = 4–5), # *p* < 0.05, ## *p* < 0.01 versus sham group; * *p* < 0.05, ** *p* < 0.01 versus UUO group.

To examine the effect of ISO treatment on UUO-induced collagen fibrils in the obstructed kidneys 14 days after the UUO operation, we utilized Masson’s trichrome staining, which revealed that UUO injury induced a significant deposition of collagen fibrils (blue area) in the renal interstitium compared to the sham group. The ISO and enalapril groups showed decreased collagen fibril deposition in the renal interstitium compared to the UUO group (Figure 2A,B). It suggested that ISO attenuates collagen deposition in the renal interstitium of obstructed kidneys. To evaluate whether ISO affects EMT formation and ECM deposition, we analyzed the expression and distribution of α-SMA and fibronectin using immunohistochemistry staining on day 14 after the UUO operation. It indicated that both ISO and enalapril decreased the expression of α-SMA and fibronectin (Figure 2C–F). Additionally, these protective effects of ISO were more potent than those of enalapril. These preliminary observations were confirmed by Western blot analysis, which suggested that ISO significantly reduced the expression of α-SMA and fibronectin and increased the expression of E-cadherin (Figure 3A–D).

These observations suggest that ISO mitigated renal fibrosis by inhibiting EMT and the deposition of ECM.

### 3.2. ISO Downregulates Renal TGF-β1 Levels and Suppresses TGF-β1-Induced EMT in Cultured Renal Tubular Cells

**Figure 4:**
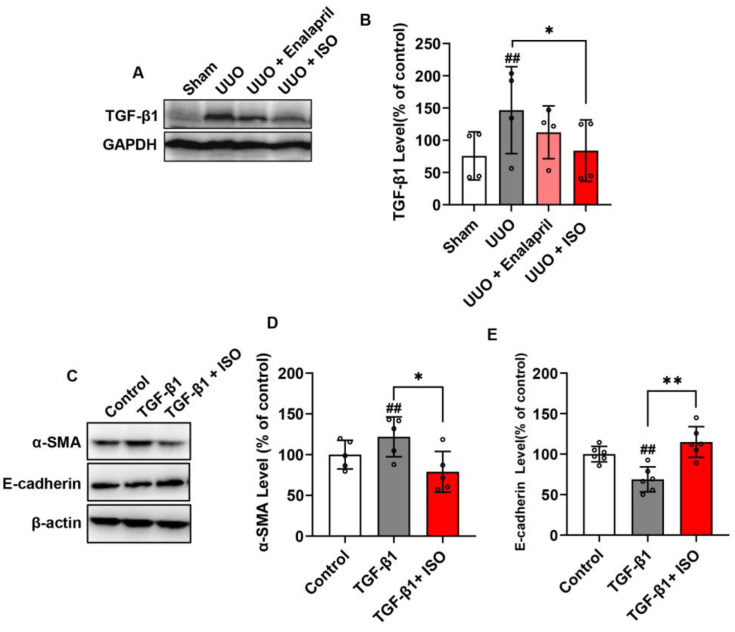
ISO downregulates the TGF-β1 level in kidney tissue and suppresses TGF-β1-induced fibrosis in NRK-52E cells. Kidney tissue lysates were assayed for TGF-β1 expression by Western blot (**A**), and the quantitative analysis of the relative densitometry of TGF-β1 is shown in (**B**). The cells were preincubated with ISO (25 μM) for 1 h, then stimulated with TGF-β1 (2 ng/mL) for 48 h. Cellular lysates were extracted and subjected to Western blot analysis for the levels of α-SMA and E-cadherin (**C**), and the quantitative analyses of the relative densitometry of these proteins are presented in (**D**,**E**). Data shown are mean ± SE (*n* = 4–6), ## *p* < 0.01 versus control group; * *p* < 0.05, ** *p* < 0.01 versus the TGF-β1 group.

To determine the potential therapeutic mechanisms of ISO, we examined the renal levels of TGF-β1, a well-known cytokine underlying kidney fibrosis. Western blot analysis of renal lysates revealed that UUO elevated TGF-β1, whereas ISO treatment significantly downregulated TGF-β1 expression (Figure 4A,B). It suggested that ISO downregulated renal TGF-β1 expression.

To determine whether ISO also interfered with TGF-β1 signaling, we established a model of TGF-β1-initiated EMT in the renal tubular epithelial cell line NRK-52E. Stimulation of NRK-52E cells with TGF-β1 resulted in increased α-SMA (a mesenchymal marker) and loss of E-cadherin (an epithelial marker), indicating induction of EMT. ISO treatment, however, reversed these effects, as evidenced by decreased α-SMA and increased E-cadherin expression (Figure 4C–E). These results indicated that ISO inhibited TGF-β1-production in vivo and interfered with the TGF-β1-mediated EMT pathway in cultured tubular epithelial cells.

### 3.3. ISO Upregulated H_2_S Levels and H_2_S-Producing Enzymes Both In Vivo and In Vitro

**Figure 5:**
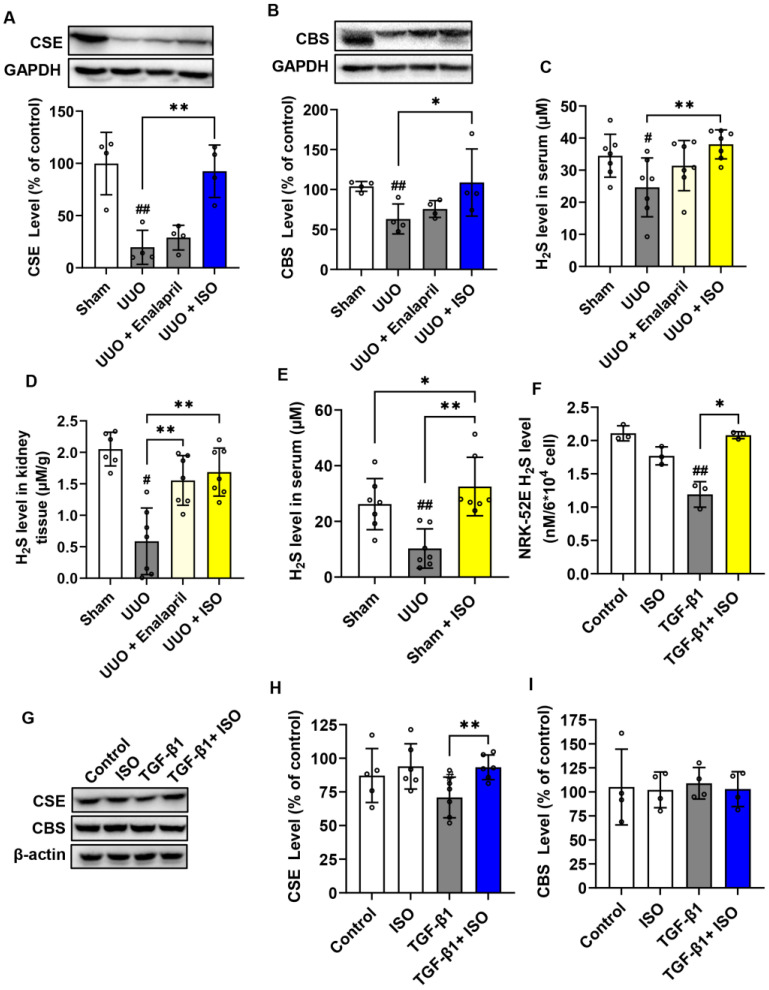
ISO upregulates H_2_S-producing enzymes and endogenous H_2_S levels both in vitro and in vivo. Kidney tissue lysates were assayed for the levels of (**A**) CSE and (**B**) CBS by Western blot. The quantitative relative densitometric analyses of CSE and CBS are presented in (**A**,**B**), *n* = 4. ISO treatment of serum H_2_S levels (**C**) and kidney tissue H_2_S production (**D**) 14 days after UUO operation, and serum H_2_S levels 14 days after sham operation (**E**) were analyzed by the methylene blue method, *n* = 7. ISO (25 μM) preincubation of H_2_S production (**F**) of TGF-β1-induced NRK-52E cells as measured by the methylene blue method. Cellular lysates were assayed for the level of CSE and CBS (**G**) by Western blot, and the quantitative relative densitometric analyses of CSE and CBS are shown in (**H**,**I**), *n* = 4. Data shown are mean ± SE (*n* = 4–7), # *p* < 0.01, ## *p* < 0.01 versus sham or control group; * *p* < 0.05, ** *p* < 0.01 versus UUO or TGF-β1 group.

The question naturally arose as to what the molecules mediating the antioxidative actions of ISO could be. We thought H_2_S could be a potential candidate. To explore this possibility, we first examined the effects of ISO on the H_2_S-synthesizing enzymes and H_2_S levels. As shown in Figure 5, the results showed that UUO injury downregulated the expression of both CSE and CBS in obstructed kidneys, whereas ISO treatment markedly upregulated both. Enalapril had no effect (Figure 5A,B). Consistent with the level of H_2_S-synthesizing enzymes, the levels of H_2_S in serum and kidney were significantly reduced, which could be substantially reversed by ISO (Figure 5C,D). Surprisingly, we found that the serum H_2_S levels of the sham + ISO group were higher than those of the Sham group. These results suggested that ISO increased the H_2_S-synthesizing enzymes and endogenous H_2_S levels in vivo.

To further confirm the effect of ISO on endogenous H_2_S levels, we investigated this in vitro using NRK-52E cells. Previous studies have demonstrated that TGF-β1 plays a profibrotic role [18]. We, therefore, used a TGF-β1-induced cell fibrotic model in NRK-52E cells. It was found that TGF-β1 decreased the H_2_S level, while ISO stimulation increased it (Figure 5F). Analysis of H_2_S-producing enzymes showed that TGF-β1 reduced CSE expression. ISO reversed this effect without affecting CBS expression (Figure 5G–I). It suggested that the reduced H_2_S-synthesizing enzyme level and H_2_S generation in the kidney of the profibrotic models could be prevented by ISO.

**Figure 6:**
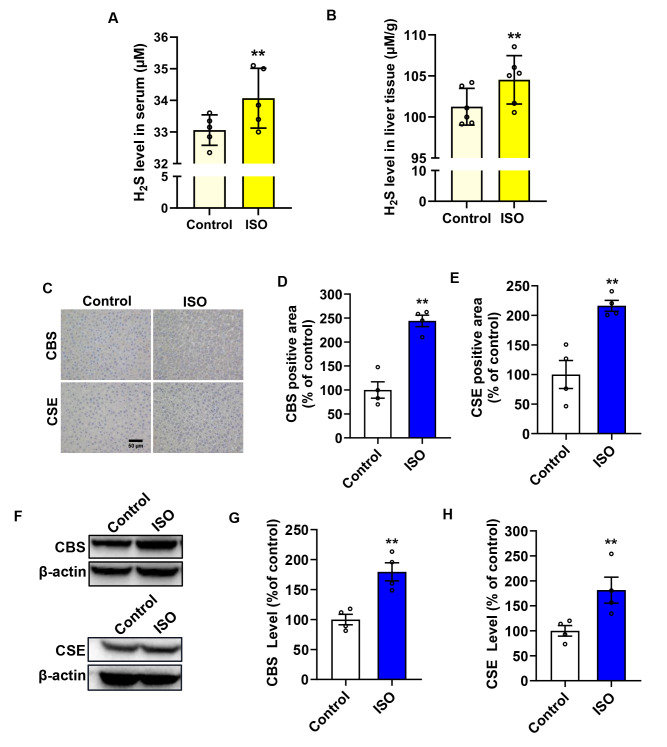
ISO enhances the endogenous level of H_2_S from the liver. ISO treatment affected serum H_2_S levels (**A**) and liver tissue H_2_S production (**B**) in normal rats at 7 days, analyzed by the methylene blue method, *n* = 6. Representative immunohistochemistry images showing the expression of CBS and CSE are presented (**C**), and the semiquantitative analyses are shown in (**D**,**E**), *n* = 6. Liver tissue lysates were assayed for levels of CBS and CSE (**F**) by Western blot, and the quantitative analyses of the relative densitometric measurements of CBS and CSE proteins are displayed in (**G**,**H**), *n* = 4. Data shown are mean ± SE (*n* = 4–6), ** *p* < 0.01 versus the control group.

We then proceeded to determine the primary sources of H_2_S induced by ISO. Given that liver-derived H_2_S has been shown to be a significant source of systemic (circulating) H_2_S [19], we investigated whether the liver is involved in the production of H_2_S. Figure 6A shows that ISO increased the serum H_2_S level in rats. Similarly, liver tissue H_2_S production followed the same trend, and surprisingly, it was approximately 100 times higher than that previously detected in kidney tissue (Figure 6B). Further analysis of the H_2_S-producing enzymes revealed that ISO increased the distribution and expression of CBS and CSE (Figure 6C–E). Western blot analysis showed similar results (Figure 6F–H). These results indicated that ISO primarily enhances the endogenous level of H_2_S from the liver.

**Figure 7:**
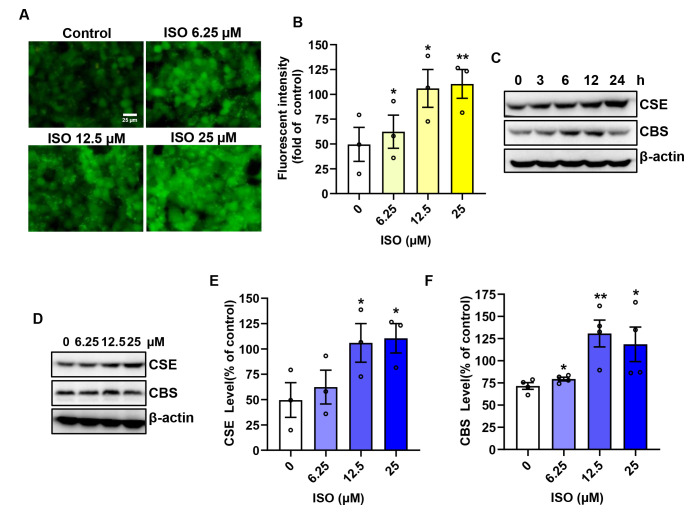
ISO increases endogenous H_2_S production in a concentration-dependent manner in HepG2 cells. (**A**,**B**) Effects of ISO on the H_2_S level in HepG2 cells. Cells were pretreated with various concentrations of ISO (6.25~50 µM) for 24 h, followed by exposure to a 5 µM H_2_S detection probe, Hsip-1 DA, for 30 min. The fluorescence in live cells was captured using a fluorescent microscope (**A**). Subsequently, the intensity of the fluorescence was quantified (**B**). (**C**–**F**) Effects of ISO on the protein levels of CBS and CSE in HepG2 cells. Cells were pretreated with 25 µM ISO for varying durations, and cellular lysates were subjected to Western blot analysis for CBS and CSE levels (**C**). Cells pretreated with various concentrations of ISO for 24 h were analyzed for CBS and CSE expression in the same manner as mentioned above (**D**). The densitometric quantification of CBS and CSE is shown in (**E**,**F**). Data shown are mean ± SE, *n* = 4, * *p* < 0.05 versus control, ** *p* < 0.01 versus control.

Exposing HepG2 cells to different concentrations of ISO showed that ISO induced H_2_S in a concentration-dependent manner, as evidenced by the fluorescence in live cells (Figure 7A) and the intensity of the fluorescence (Figure 7B). We also examined the effect of ISO on the expression of CBS and CSE. Figure 7C shows that 25 µM ISO increased the expression of CSE in a time-dependent manner. Additionally, the levels of CBS were elevated before 12 h but decreased after 12 h. Further analysis of the ISO concentration curves for CBS and CSE expression revealed that ISO led to an increase in CSE expression in a concentration-dependent manner, with CBS initially increasing and then decreasing within the concentration range from 6.25 to 50 µM (Figure 7D–F). These results demonstrate that ISO induces the endogenous level of H_2_S in HepG2 cells.

### 3.4. H_2_S Contributes to the Suppressive Effects of ISO on TGF-β1-Induced Fibrosis

**Figure 8:**
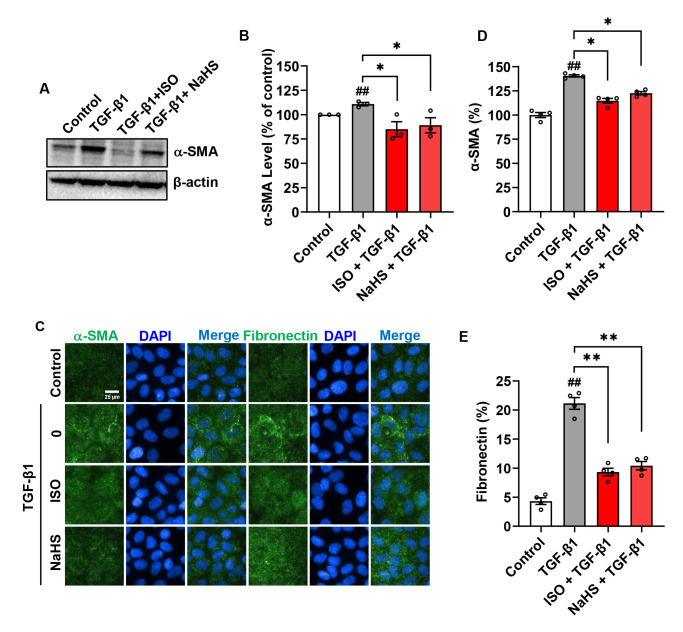
ISO and the H_2_S donor, NaHS, suppress TGF-β1-induced fibrosis in NRK-52E cells. (**A**) Effect of ISO on TGF-β1-induced α-SMA in NRK-52E cells. The cells were pretreated with 25 µM ISO or 500 µM NaHS for 1 h, then exposed to TGF-β1 for 24 h. Cellular lysates were subjected to Western blot analysis for α-SMA. The densitometric analysis of α-SMA is shown in (**B**), *n* = 3. (**C**) Immunofluorescence staining for α-SMA or fibronectin was conducted as previously described, followed by fluorescence microscopy imaging of NRK-52E cells. In the images, α-SMA and fibronectin are shown in green and DAPI in blue. The cellular intensities of fluorescence of α-SMA (**D**) and fibronectin (**E**) were quantified and expressed as a fold of control, *n* = 4. Data shown are mean ± SE, *n* = 3–4, ## *p* < 0.01 versus control, * *p* < 0.05, ** *p* < 0.01 versus TGF-β1.

To determine the role of elevated H_2_S in the antifibrotic action of ISO, we used an H_2_S donor, NaHS. Similar to the action of ISO, NaHS also inhibited α-SMA expression in Western blot analysis and IF staining (Figure 8A–D). Additionally, IF staining revealed that NaHS and ISO also inhibited the expression of fibronectin (Figure 8C–E). These observations indicate that elevated H_2_S may contribute to the suppressive actions of ISO on TGF-β1-induced fibrosis.

### 3.5. ISO Relieves TGF-β1-Induced Oxidative Stress through Disruption of the keap1-Nrf2 Interaction in NRK-52E Cells

**Figure 9:**
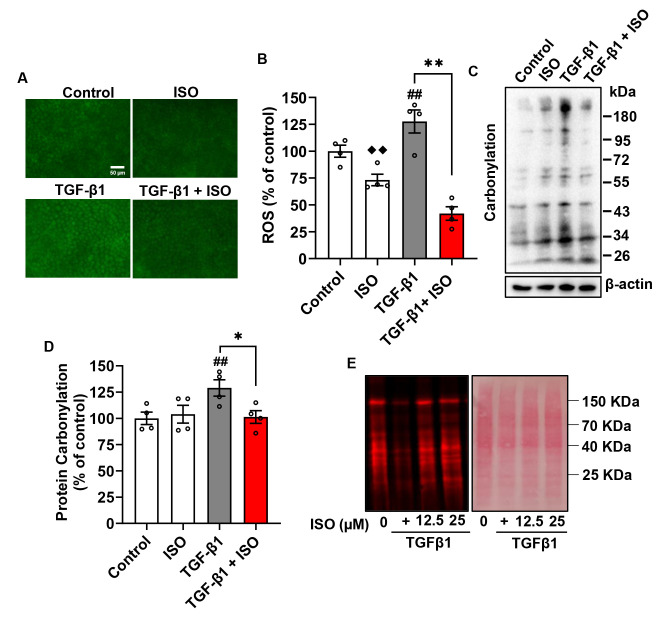
ISO inhibits TGF-β1-induced oxidative stress. (**A**) Effect of ISO on TGF-β1-induced oxidative stress in NRK-52E cell. Cells were preincubated with 25 µM ISO for 1 h, then exposed to TGF-β1 for 24 h. Subsequently, the intracellular ROS levels were assayed using a kit from Enzo, the intracellular fluorescence was captured using a fluorescent microscope (**A**) and the intensity of fluorescence was quantified (**B**). (**C**,**D**) Effect of ISO on TGF-β1-induced protein carbonylation. Cells were treated the same as above and cellular lysates were subjected to Western blot analysis (**C**). Densitometric analysis of protein carbonylation is shown in (**D**). (**E**) Effect of ISO on free -SH activity in TGF-β1-induced NRK-52E cell. Cells were treated with varying concentrations of ISO for 1 h, followed by TGF-β1 for 24 h. Free -SH was detected by maleimide labeling assay (**E**, left panel). Equal loading of protein was confirmed by Ponceau S staining (**E**, right panel). Data shown are mean ± SE, *n* = 4, ## *p* < 0.01 versus control; ♦♦ *p* < 0.01 versus control; * *p* < 0.01, ** *p* < 0.01 versus TGF-β1.

Given that TGF-β1-induced excessive accumulation of ROS is the primary reason for renal fibrosis [20], we investigated whether ISO altered oxidative status. For this purpose, we examined the effect of TGF-β1 on intracellular ROS and oxidative stress markers in cultured tubular epithelial cells. It was found that TGF-β1 stimulated ROS production, as revealed by the increased fluorescent intensity after the addition of the ROS probe. This effect of TGF-β1 was completely reversed by ISO (Figure 9A,B). Consistently, ISO also obviously lowered the TGF-β1-induced protein carbonylation (Figure 9C,D), and protein thiol levels (Figure 9E). As a crucial component of the cell’s antioxidant defense system, protein thiol is highly sensitive to changes in cellular redox status [21]. These results indicate that ISO counteracted TGF-β1-induced oxidative stress in renal tubular epithelial cells.

**Figure 10:**
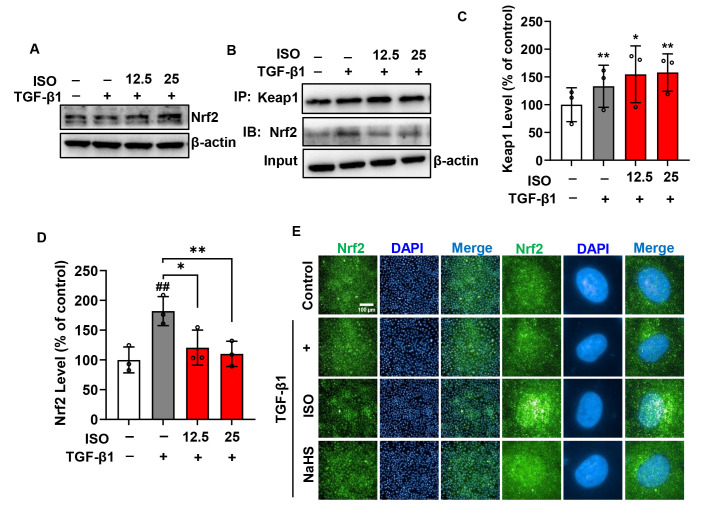
ISO disrupts keap1-Nrf2 interaction in TGF-β1-induced NRK-52E cells. (**A**) Effect of ISO on Nrf2 expression in TGF-β1-induced NRK-52E cells. Cells were preincubated with various concentrations of ISO for 1 h, followed by exposure to TGF-β1 for 48 h. Cellular lysates were subjected to Western blot analysis of Nrf2 (**A**). Effect of ISO on Keap1-Nrf2 interaction in TGF-β1-induced NRK-52E cells. Cells were treated as described above, followed by coimmunoprecipitation of Keap1-Nrf2 using protein A/G beads and subsequent Western blot analysis (**B**). Densitometric analysis of Keap1 (**C**) and Nrf2 (**D**) was conducted. (**E**) Immunofluorescence analysis of Nrf2 in NRK-52E Cells. NRK-52E cells were stimulated with 25 µM ISO or 500 µM NaHS for 1 h, followed by the addition of TGF-β1 for 24 h. After stimulation, the cells underwent immunofluorescence staining for Nrf2 and were imaged using fluorescence microscopy. In the images, Nrf2 is shown in green and DAPI in blue. Data shown are mean ± SE, *n* = 3, ## *p* < 0.01 versus control; * *p* < 0.05, ** *p* < 0.01 versus TGF-β1.

The Keap1-Nrf2 signaling pathway plays a crucial role in regulating the redox state. Therefore, we tested the possibility of participation in this pathway. It showed that different concentrations of ISO upregulated Nrf2 expression in TGF-β1-stimulated NRK-52E cells (Figure 10A). To determine the effect of ISO on Keap1-Nrf2 interaction, we performed a coimmunoprecipitation analysis. It was found that TGF-β1 elevated Keap1 levels and enhanced the bonds between Keap1 and Nrf2. ISO also increased Keap1 levels but diminished the bonds with Nrf2, reducing the interaction between Keap1 and Nrf2 (Figure 10B–D). Subsequently, we performed immunofluorescence analysis and found that ISO facilitated the translocation of Nrf2 into the cell nucleus. Similar results were observed when cells were stimulated with NaHS. However, ISO displayed greater efficacy in promoting Nrf2 translocation compared to NaHS.

These observations indicated that ISO inhibited oxidative stress by disrupting Keap1-Nrf2 interaction and promoting the translocation of Nrf2 into the nucleus in TGF-β1-stimulated NRK-52E cells.

### 3.6. ISO Induces Keap1 Sulfhydration in TGF-β1-Stimulated NRK-52E Cells

**Figure 11:**
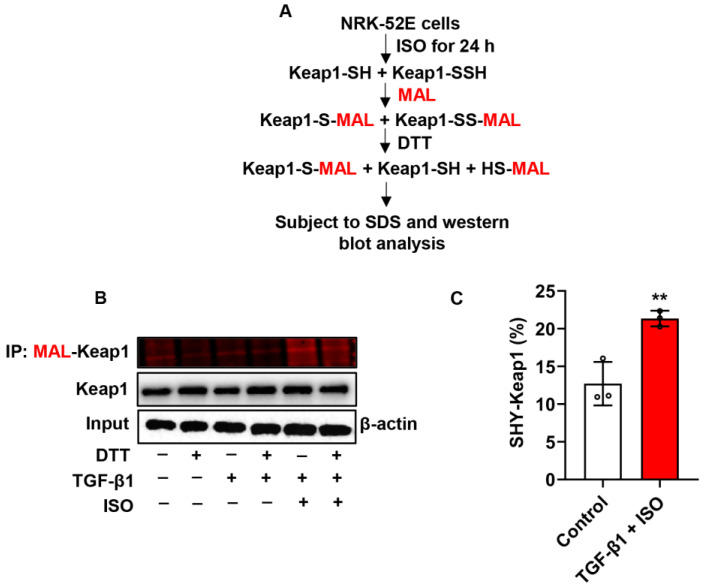
ISO induces Keap1 sulfhydration. (**A**) Schematic representation of the method used for detecting Keap1 sulfhydration. In this assay, cells were stimulated with ISO for 1 h, which was followed by the addition of TGF-β1 for 24 h. Afterward, keap1 was immunoprecipitated and reacted with Alexa Fluor^®^ 680 C2 maleimide (red) to label both -SSH and -SH Cys in Keap1. After removing excess maleimide, DTT was added to cleave the disulfide bonds, resulting in the loss of the fluorescent signal from sulfhydrated (-SSH) but not from unsulfhydrated Keap1. The sulfhydration of Keap1 can be quantified by the decrease in fluorescence. (**B**) The Maleimide-labelled samples were analyzed by SDS-PAGE. (**C**) Quantitative calculation of sulfhydrated Keap1 by ISO. Data shown are mean ± SE, *n* = 3, ** *p* < 0.01 versus control.

Because H_2_S has a reductive activity that maintains protein cysteine (Cys) residues in the reduced state and induces protein sulfhydration, a post-translational modification occurs when H_2_S adds additional sulfur to the sulfhydryl (-SH) groups of Cys residues to form -SSH groups in proteins, resulting in changes to their structure and function [22]. Recent studies have shown that the sulfhydration of Keap1 could disrupt the Keap1-Nrf2 interaction [23,24]; therefore, we explored these possibilities. The maleimide assay was used to detect sulfhydrated Keap1. It revealed that ISO significantly increased the sulfhydration of Keap1, as evidenced by the loss of fluorescent signal following DTT treatment. This result confirms the presence of sulfhydration of Keap1 (Figure 11A–C). Together with literature reports, these results indicated that ISO disrupts Keap1-Nrf2 interaction via the sulfhydration of Keap1 in TGF-β1-stimulated NRK-52E cells.

## 4. Discussion

ISO is a flavonoid found abundantly in various plants, and it boasts diverse pharmacological properties [11]. This study establishes ISO’s ability to inhibit renal fibrosis in obstructive nephropathy, primarily by increasing endogenous H_2_S levels both in vivo and in vitro. Given the established antifibrotic properties of H_2_S [9,10], its induction by ISO emerges as a crucial mechanism underlying ISO’s therapeutic effects. The UUO model serves as a valuable tool for studying renal fibrosis progression without the confounding factors of exogenous toxins or a uremic environment [15]. Our research, consistent with previous studies, demonstrates that UUO induces characteristic fibrotic changes, including reduced renal size and weight, increased cortical thickness, EMT formation, and ECM deposition. During the in vivo study, we administered 10 mg/kg/day and 30 mg/kg/day. Since the results obtained from the low and high concentrations were similar, we have decided to only present the findings from the low concentration in this paper. Importantly, ISO effectively counteracts these detrimental changes, highlighting its potential as a therapeutic agent against renal fibrosis.

TGF-β1 plays a central role in renal fibrosis development, driving EMT, promoting profibrotic actions in renal epithelial cells (RECs), and contributing to oxidative stress [20]. Our findings reveal that ISO effectively reduces renal TGF-β1 levels and mitigates its downstream effects. Notably, ISO inhibits TGF-β1-induced EMT and profibrotic actions in cultured RECs and demonstrates antioxidant properties by counteracting TGF-β1-induced oxidative stress. This action is particularly significant as increased reactive ROS can activate signaling pathways (e.g., ERK) that exacerbate EMT and fibrosis [20]. The ability of ISO to target both TGF-β1 and oxidative stress underscores its potential for a multifaceted approach to combatting renal fibrosis.

The question arises, how does ISO exert its antifibrotic effects? One crucial mechanism involves the regulation of H_2_S, the third endogenous gasotransmitter alongside carbon monoxide and nitric oxide. H_2_S, primarily produced by the enzymes CSE, CBS, and 3-mercaptopyruvate sulfurtransferase (3-MST), plays a vital role in physiological and pathological processes [25]. Clinical studies have reported decreased serum H_2_S levels in patients with renal fibrosis and CKD [26], and supplementing with exogenous H_2_S donors has shown promise in inhibiting renal fibrosis development [9]. Our study aligns with these findings, demonstrating that UUO-induced renal fibrosis is associated with reduced expression of H_2_S-synthesizing enzymes and decreased renal H_2_S production. Notably, TGF-β1 itself can downregulate H_2_S-synthesizing enzymes, further contributing to H_2_S deficiency and fibrosis progression [6,27].

We observed that ISO not only counteracts UUO-induced renal fibrosis but also significantly elevates H_2_S levels both locally in the kidney and systemically in the serum. Interestingly, the liver, a major H_2_S-producing organ, exhibited increased H_2_S-synthesizing enzymes upon ISO treatment, suggesting a potential contribution to the elevated serum H_2_S levels.

The antifibrotic actions of ISO mediated by H_2_S likely involve multiple mechanisms. H_2_S can directly deactivate TGF-β1, inhibit its signaling pathway, scavenge ROS, stimulate antioxidant enzyme synthesis, and counteract vasoconstrictive factors like angiotensin II [28]. Given the established role of oxidative stress in UUO-induced fibrosis, we focused on the antioxidant effects of H_2_S. H_2_S exerts its antioxidant action by directly scavenging ROS and modulating redox-related signaling pathways, including the Nrf2-Keap1 system, a crucial regulator of redox homeostasis [21]. Previous research suggests that H_2_S can modulate the Nrf2-Keap1 pathway [23,24], and ISO has been shown to increase thiol activity and potentially regulate this pathway [29]. Our findings support this hypothesis, demonstrating that ISO regulates Nrf2 expression, disrupts Nrf2-Keap1 interaction, and promotes Nrf2 nuclear translocation, ultimately leading to antioxidant and antifibrotic effects.

How does ISO disrupt the Nrf2-Keap1 interaction? This may be related to the elevated levels of H_2_S induced by ISO. Sulfhydration is the process through which H_2_S modifies cysteine thiol groups (-SH) or disulfide bonds in target proteins to produce -SSH groups. The role of H_2_S primarily involves the sulfhydration modification of target proteins, which alters their structure, activity, stability, and interactions between proteins, thus regulating intracellular signaling pathways and related biological processes [30]. Currently, several studies have demonstrated that sulfhydration of Keap1 promotes Nrf2 activation and exerts its protective action. For example, it protects cardiomyocytes from doxorubicin-induced ferroptosis [31], alleviates CCl_4_-induced liver injury [32], and suppresses diabetes-accelerated atherosclerosis [23]. We therefore wondered whether the sulfhydration of Keap1 is involved in the elevation of H_2_S by ISO. For this purpose, we conducted the maleimide assay and confirmed that ISO sulfhydrates Keap1. However, direct evidence is needed to ascertain which Cys site in Keap1 is sulfhydrated by the elevated H_2_S induced by ISO to disrupt Nrf2-Keap1 interaction. Research in this area is limited. Two studies have demonstrated sulfhydration of Keap1 at Cys151 by H_2_S, which reduces its binding ability with Keap1 [23,24]. In contrast, another study has shown that H2S sulfhydrates Keap1 at the C226–C613 disulfide bond [33]. It appears that sulfhydration of Keap1 may occur at different Cys sites depending on the cellular environment.

This study presents several significant implications. First, it establishes ISO as a promising therapeutic agent for renal fibrosis, addressing a critical unmet medical need. Second, it highlights the role of H_2_S induction as a key mechanism underlying ISO’s antifibrotic effects, potentially through H_2_S-mediated regulation of the Nrf2-Keap1 pathway. Our study also suggests that H_2_S may contribute to other pharmacological effects of ISO, such as vasodilation, anti-inflammation, and neuroprotection [11]. Finally, considering the association of H_2_S deficiency with various diseases [19], the upregulation of H_2_S-synthesizing enzymes by ISO could be exploited to develop novel strategies for these conditions.

## 5. Conclusions

In conclusion, our research demonstrates that ISO alleviates renal fibrosis by inducing endogenous H_2_S and modulating Keap1-Nrf2 interaction through Keap1 sulfhydration. This study underscores the significant role of H_2_S in mediating ISO’s antifibrotic effects and highlights the potential of H_2_S-inducing strategies for treating renal fibrosis and other H_2_S-deficient diseases.

## Data Availability

The data is contained within the article.

## Supplementary Materials

The following supporting information can be downloaded at: https://www.mdpi.com/article/10.3390/biom14101233/s1, Figure S1: The effect of ISO on renal morphology in UUO Rats; Figure S2: The effect of ISO on α-SMA, E-cadherin and Fibronectin expression in UUO Rats; Figure S3: The effect of ISO on TGF-β1 expression in UUO Rats; Figure S4: The effect of ISO on α-SMA expression in TGF-β1-induced NRK-52E cells; Figure S5: The effect of ISO on E-cadherin expression in TGF-β1-induced NRK-52E cells; Figure S6: The effect of ISO on CSE and CBS expression in UUO Rats; Figure S7: The effect of ISO on CSE and CBS expression in TGF-β1-induced NRK-52E cells; Figure S8: The effect of ISO on CSE and CBS expression in rat liver; Figure S9: The effect of ISO on CSE and CBS expression in HepG2 cells; Figure S10: The effect of ISO and NaHS on α-SMA expression in TGF-β1-induced NRK-52E cells; Figure S11: The effect of ISO on carbonylation and thiol level in TGF-β1-induced NRK-52E cells; Figure S12: The effect of ISO on Keap1 and Nrf2 interaction in TGF-β1-induced NRK-52E cells; Figure S13: ISO induces Keap1 sulfhydration in TGF-β1-induced NRK-52E cells.
