# Supplementary material for: Isorhamnetin Alleviates Renal Fibrosis by Inducing Endogenous Hydrogen Sulfide and Regulating Thiol-Based Redox State in Obstructed Kidneys
Source: Biomolecules. 2024 Sep 29;14(10):1233. doi: 10.3390/biom14101233 (PMC11506782; doi:10.3390/biom14101233)

Figure S1:The effect of ISO on renal morphology in UUO Rats

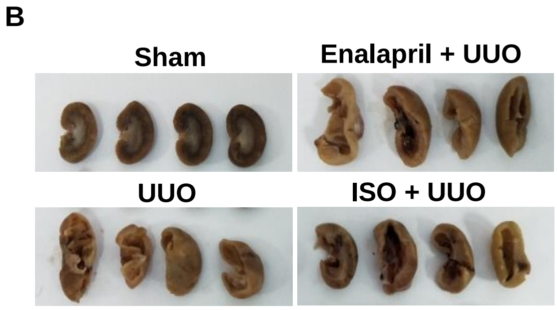

Original

Sham

UUO

Enalapril+
UUO

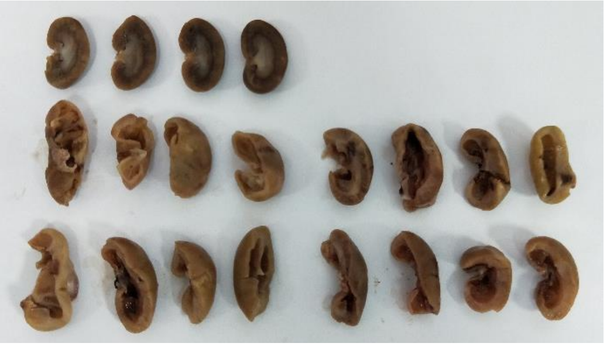

ISO 10 mg/kg/day + UUO

ISO 30 mg/kg/day + UUO

Figure S2: The effect of ISO on α-SMA, E-cadherin and Fibronectin expression in UUO Rats

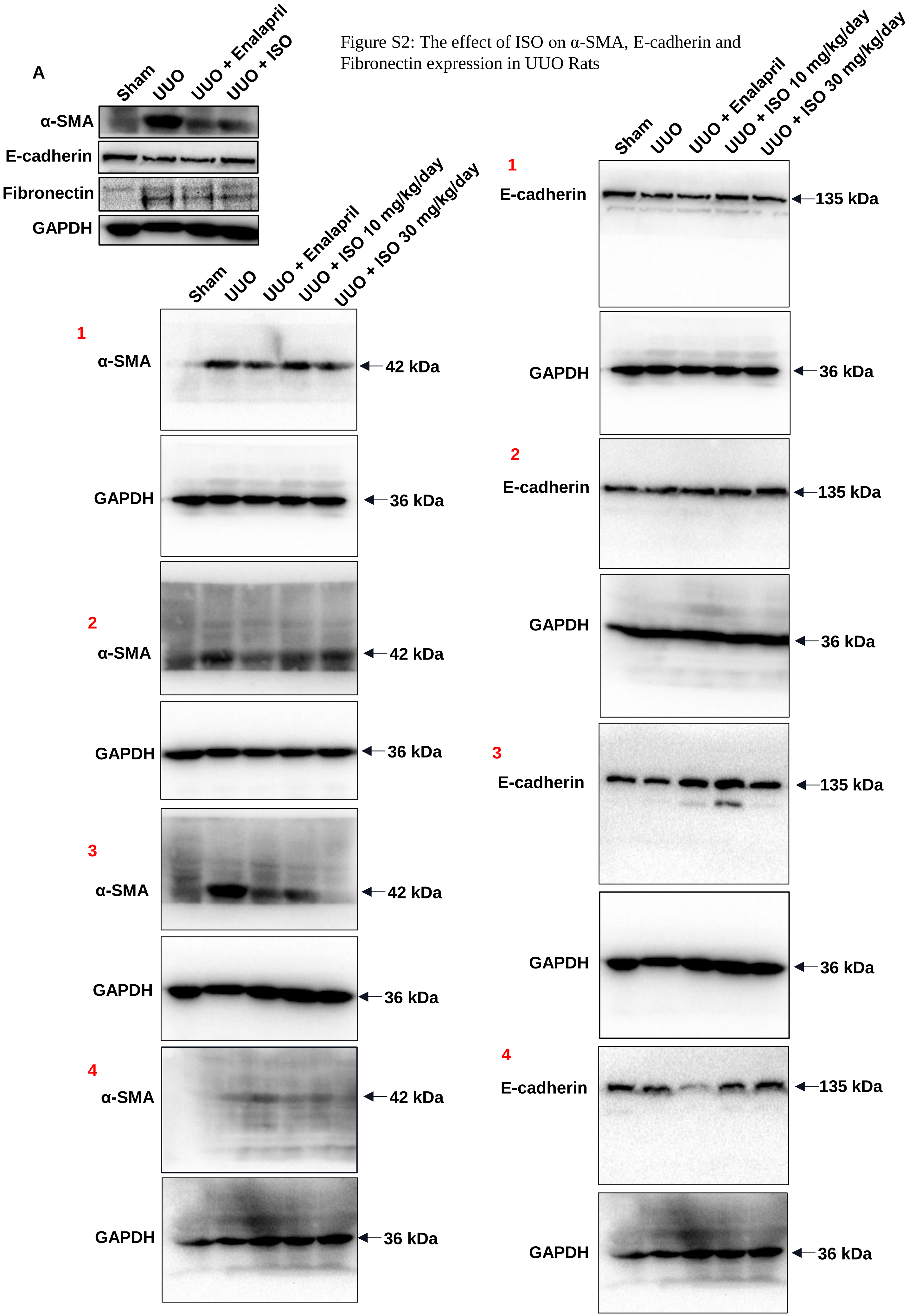

Figure S2: The effect of ISO on α-SMA, E-cadherin and Fibronectin expression in UUO Rats

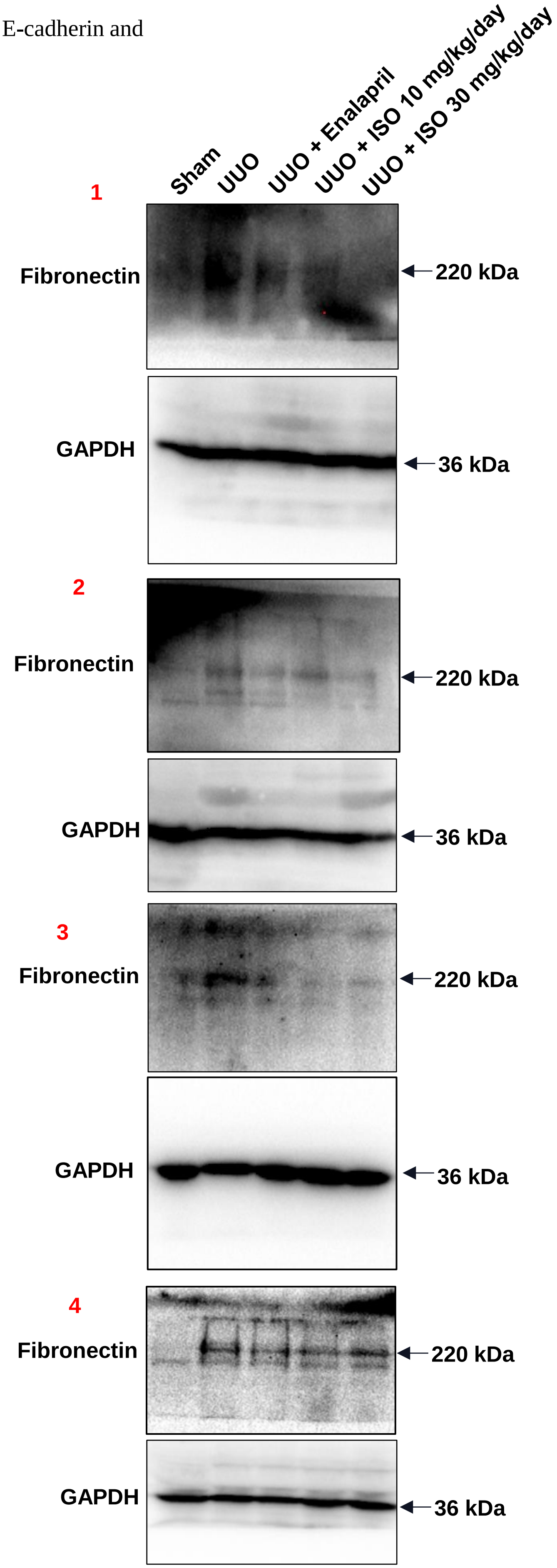

Figure S3: The effect of ISO on TGF-β1 expression in UUO Rats

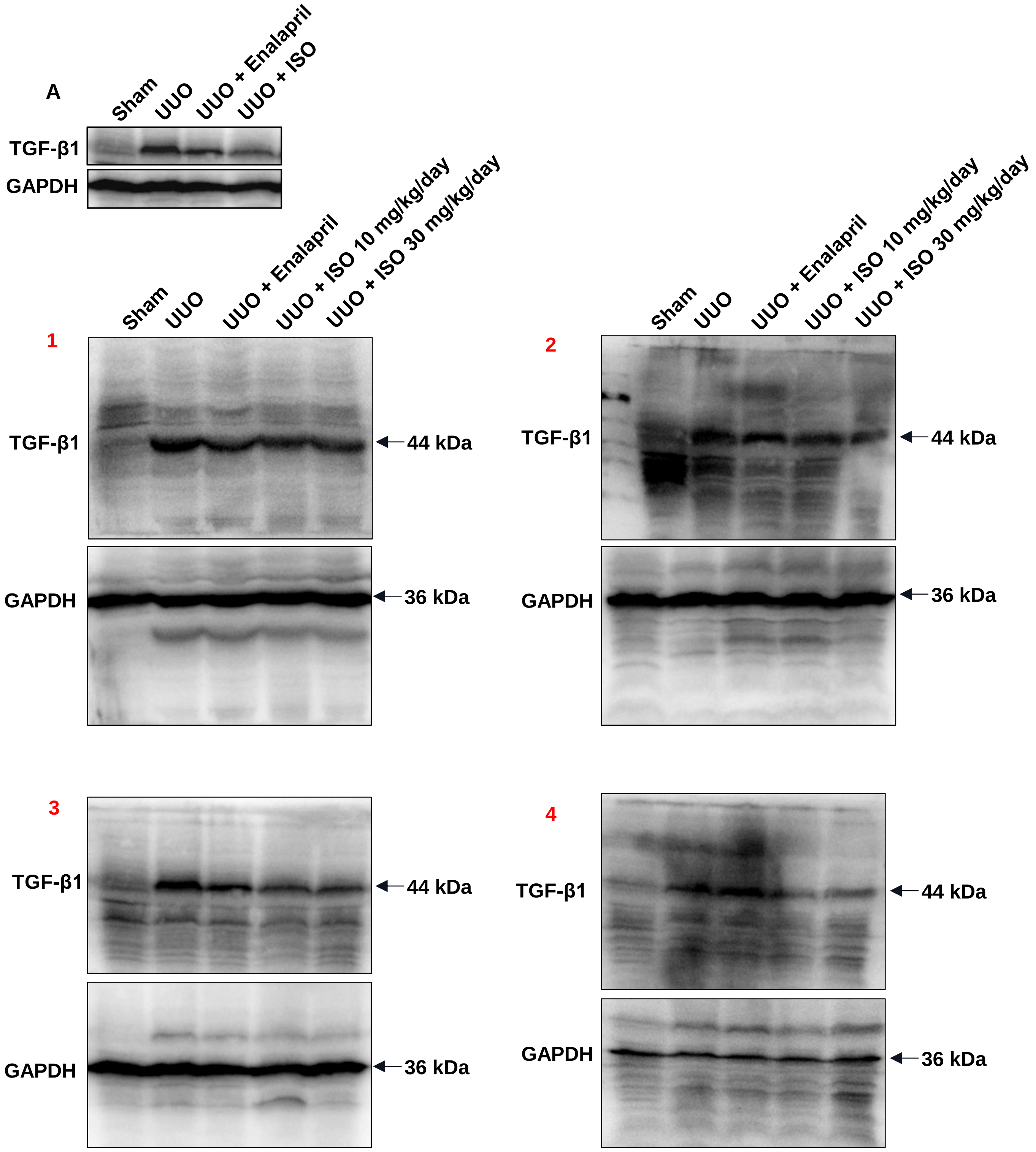

Figure S4: The effect of ISO on α-SMA expression in NRK-52E cell

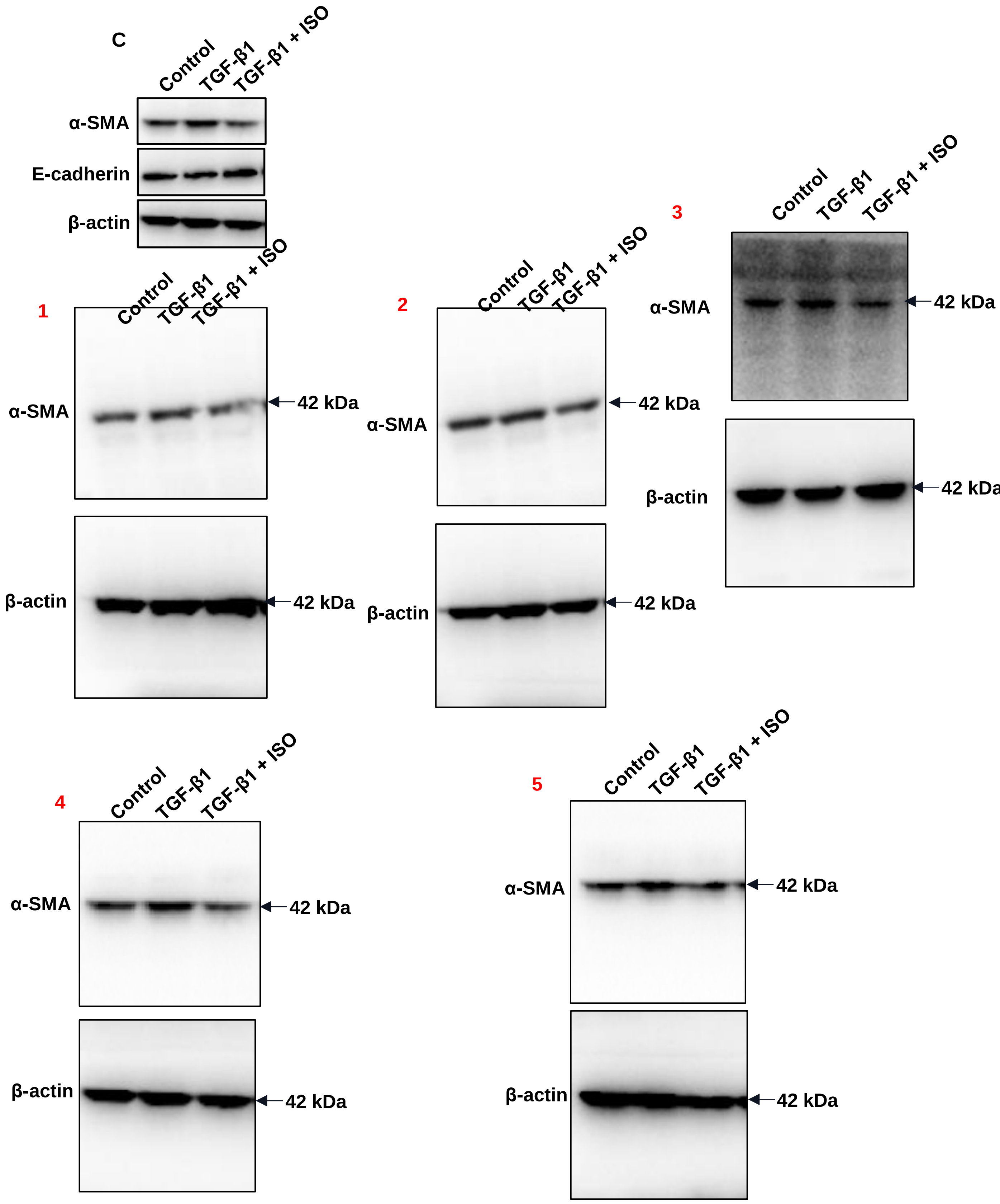

Figure S5: The effect of ISO on E-cadherin expression in TGF-β1-induced NRK-52E cells

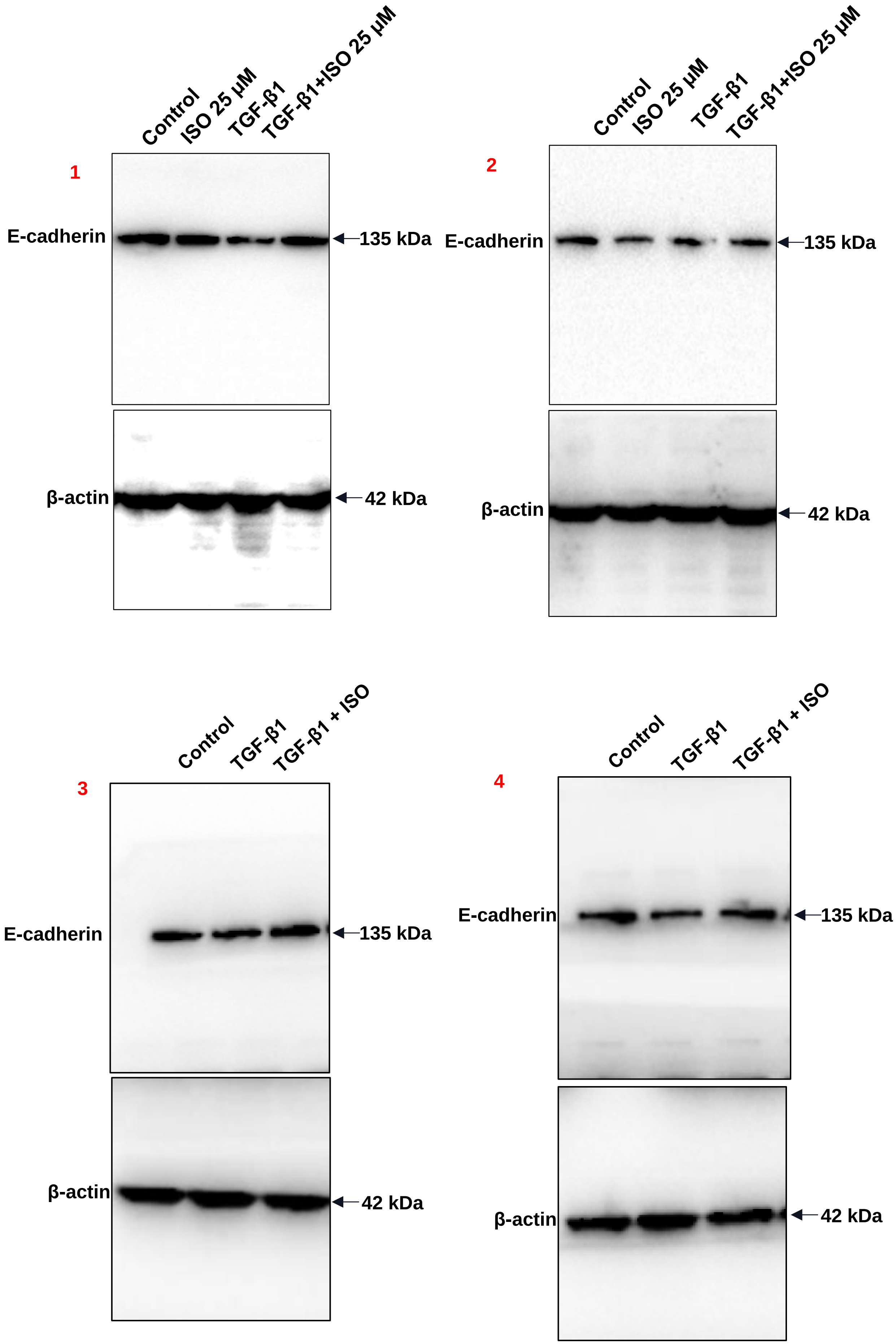

Figure S6: The effect of ISO on CSE and CBS expression in UUO Rats

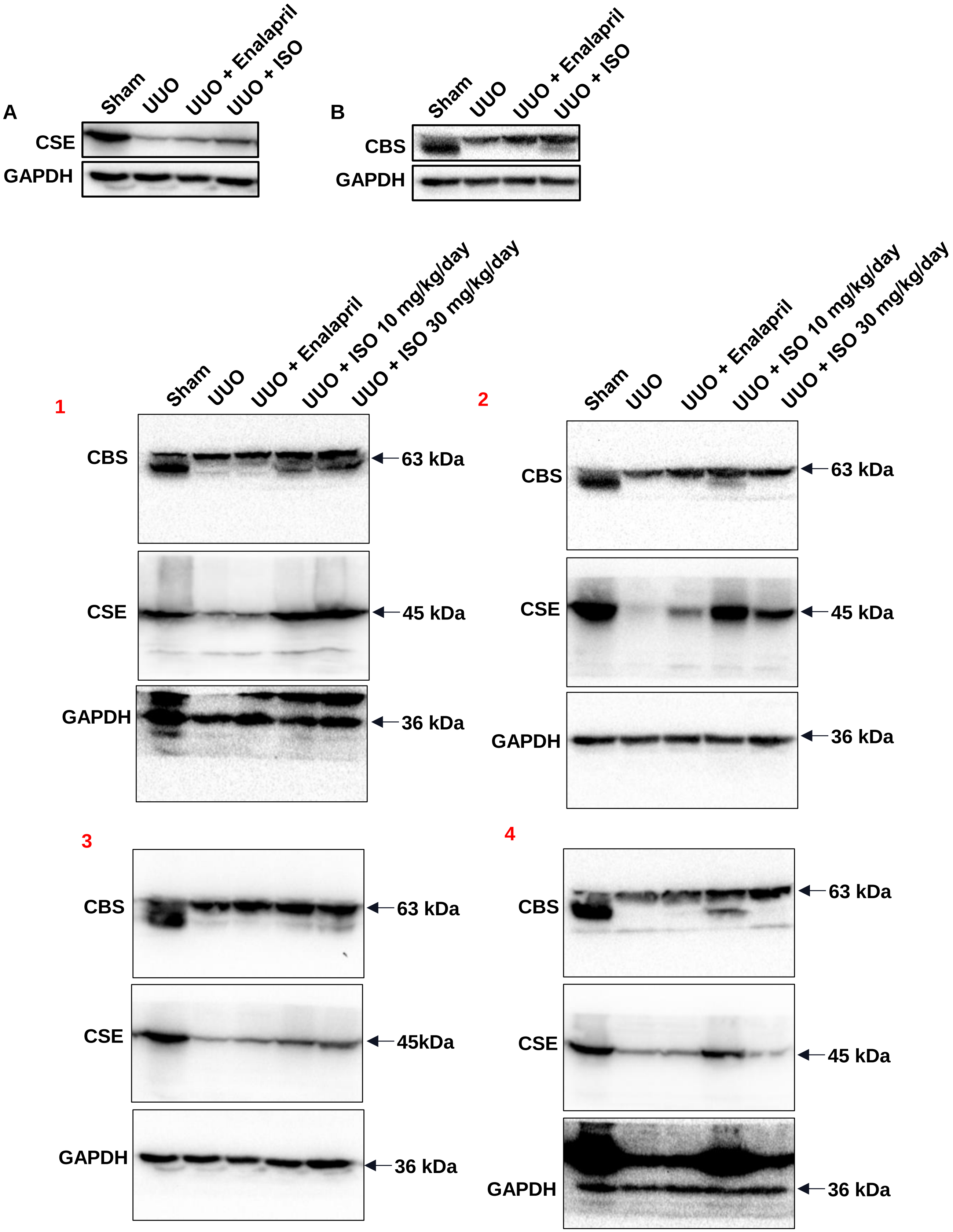

Figure S7: The effect of ISO on CSE and CBS expression in TGF-β1-induced NRK-52E cells

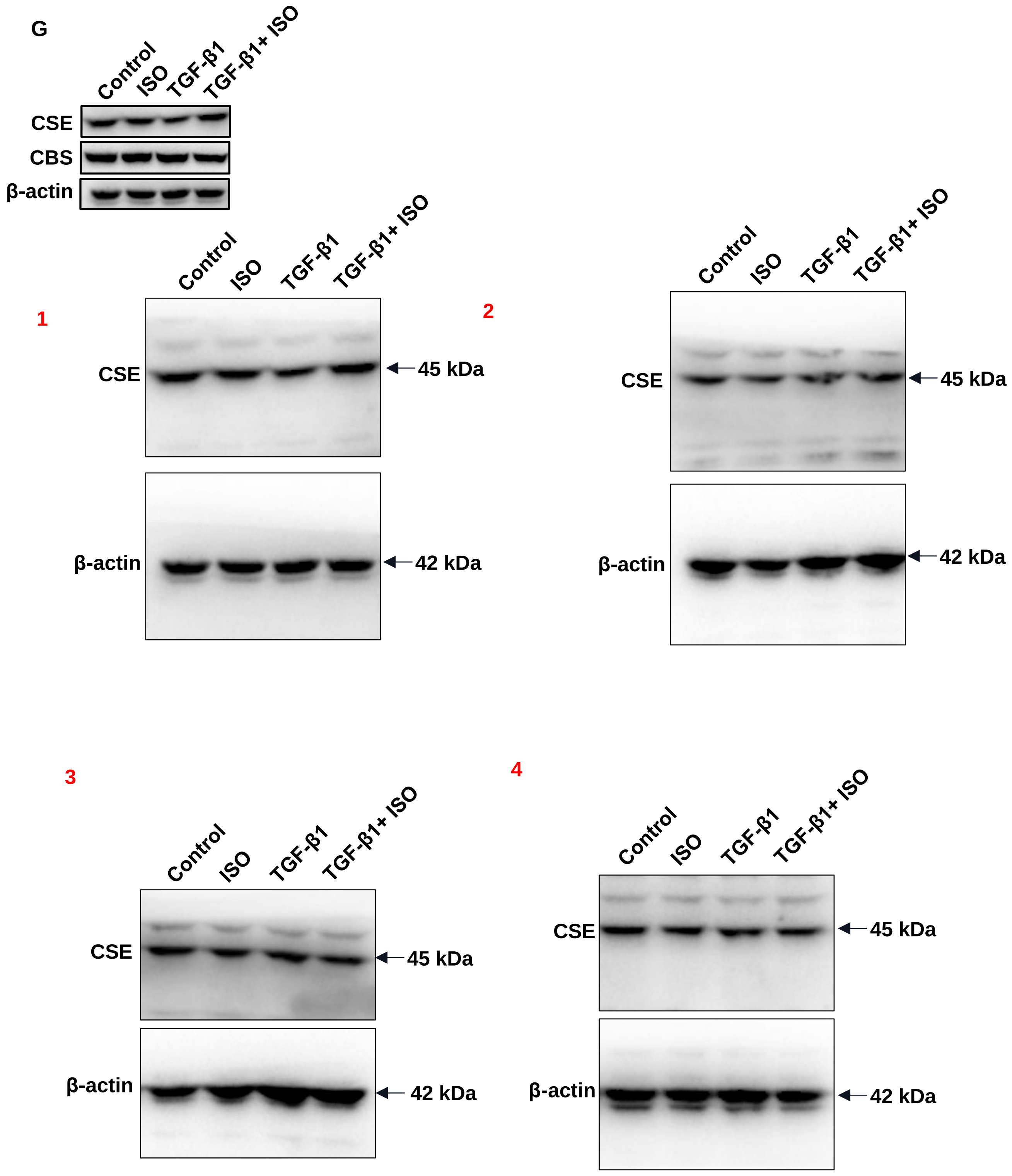

Figure S8: The effect of ISO on CSE and CBS expression in rat liver

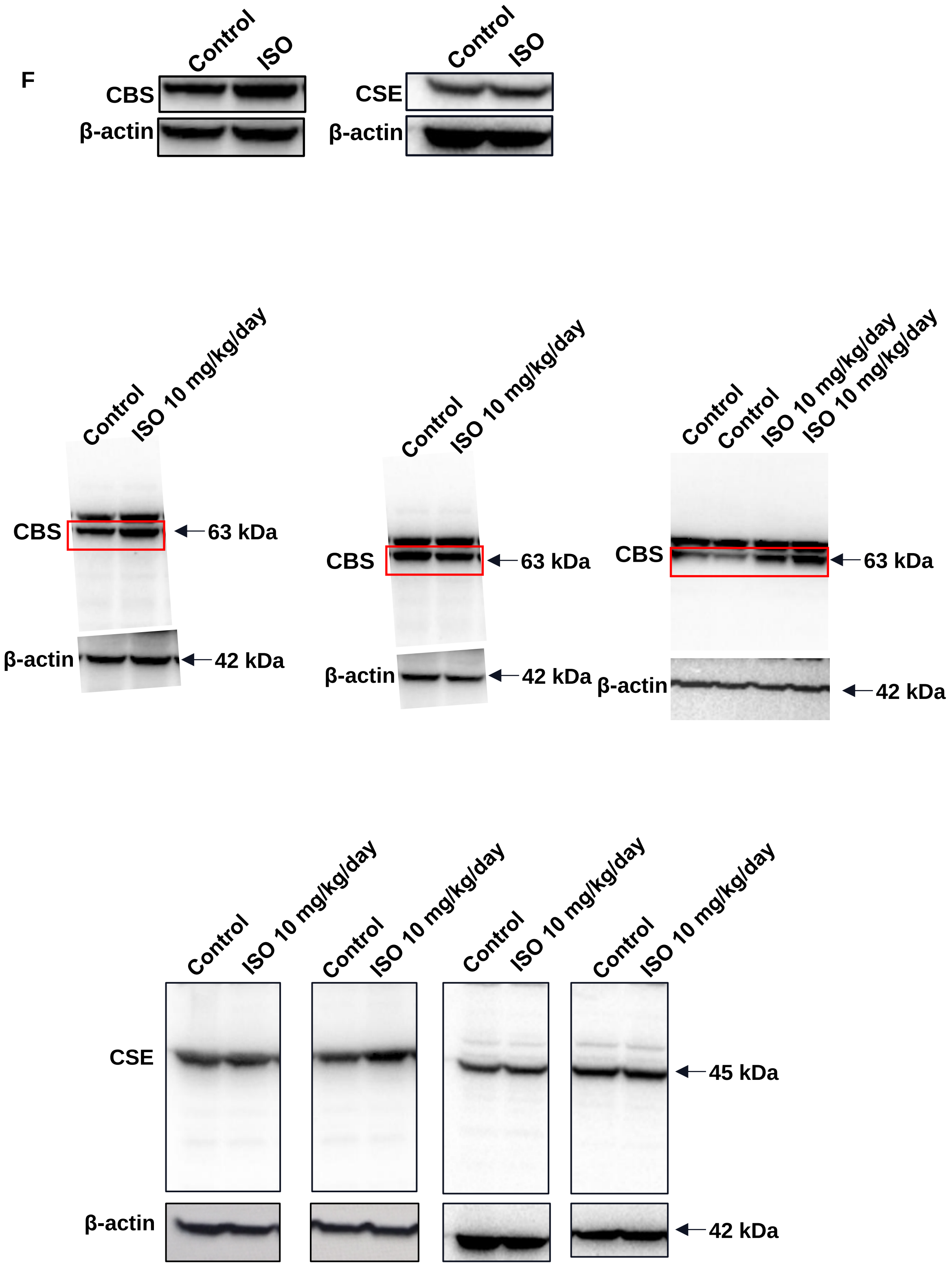

Figure S9: The effect of ISO on CSE and CBS expression in HepG2 cells

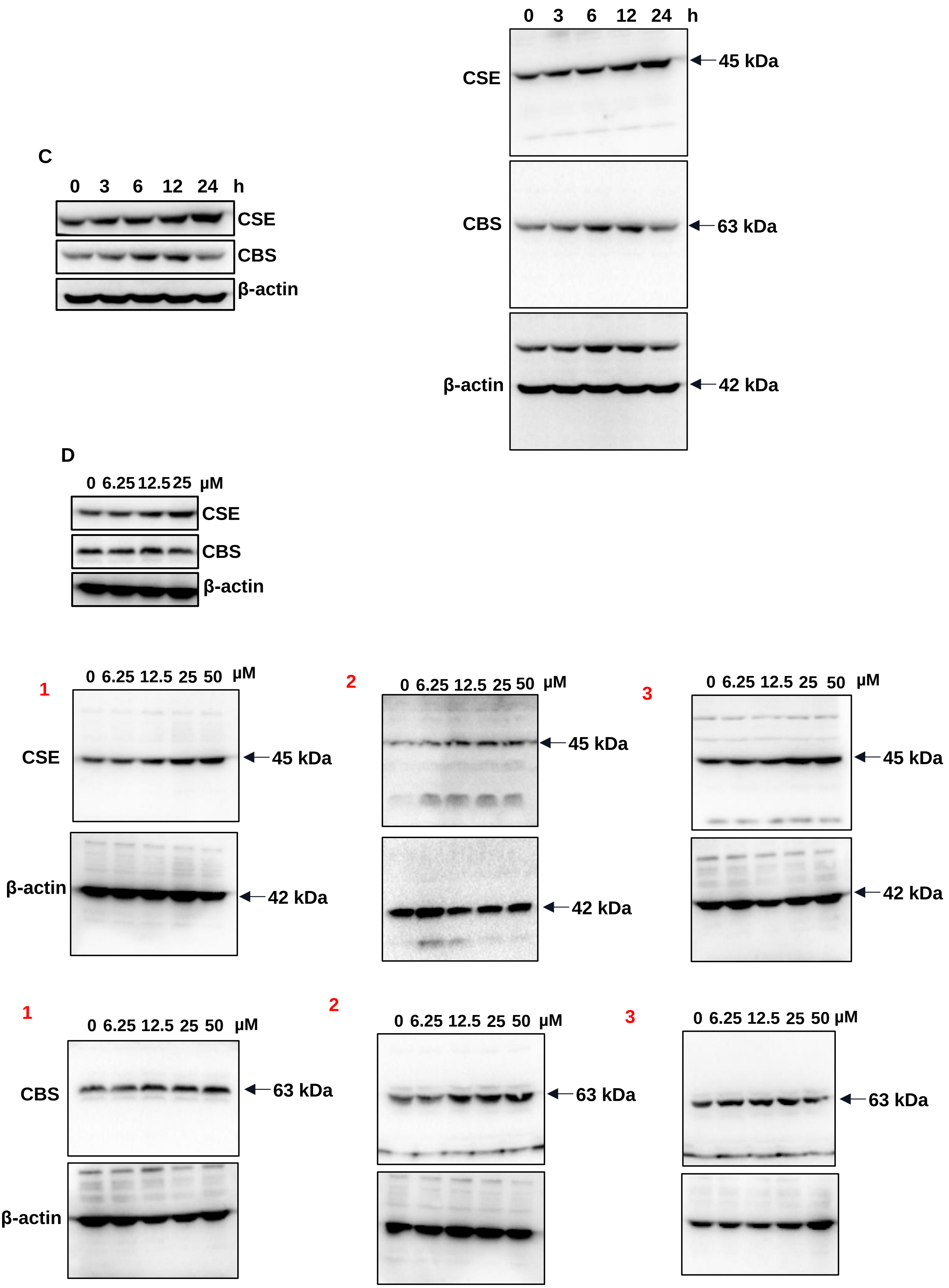

Figure S10: The effect of ISO and NaHS on α-SMA expression in TGF-β1-induced NRK-52E cells

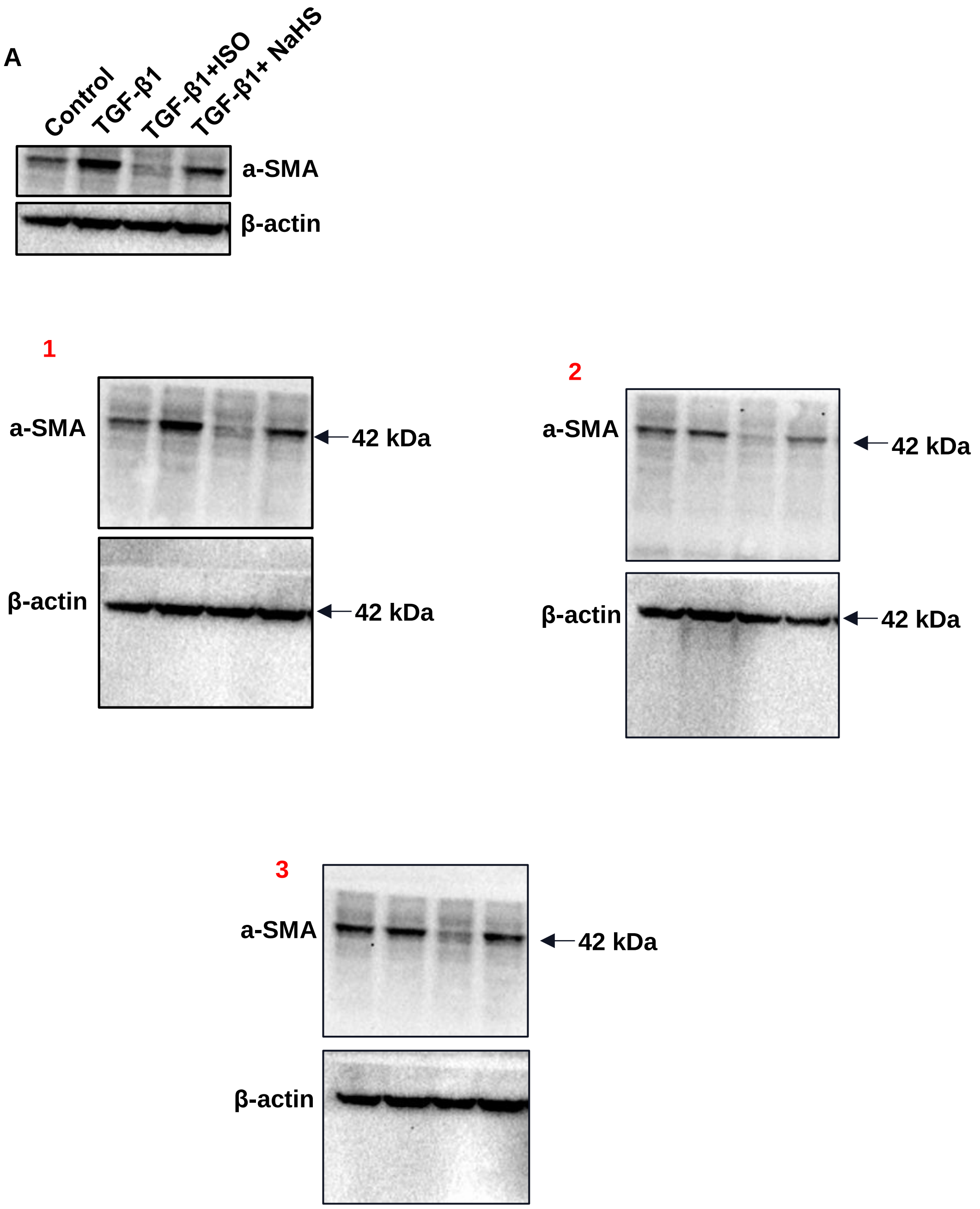

Figure S11: The effect of ISO on carbonylation and thiol level in TGF-β1-induced NRK-52E cells

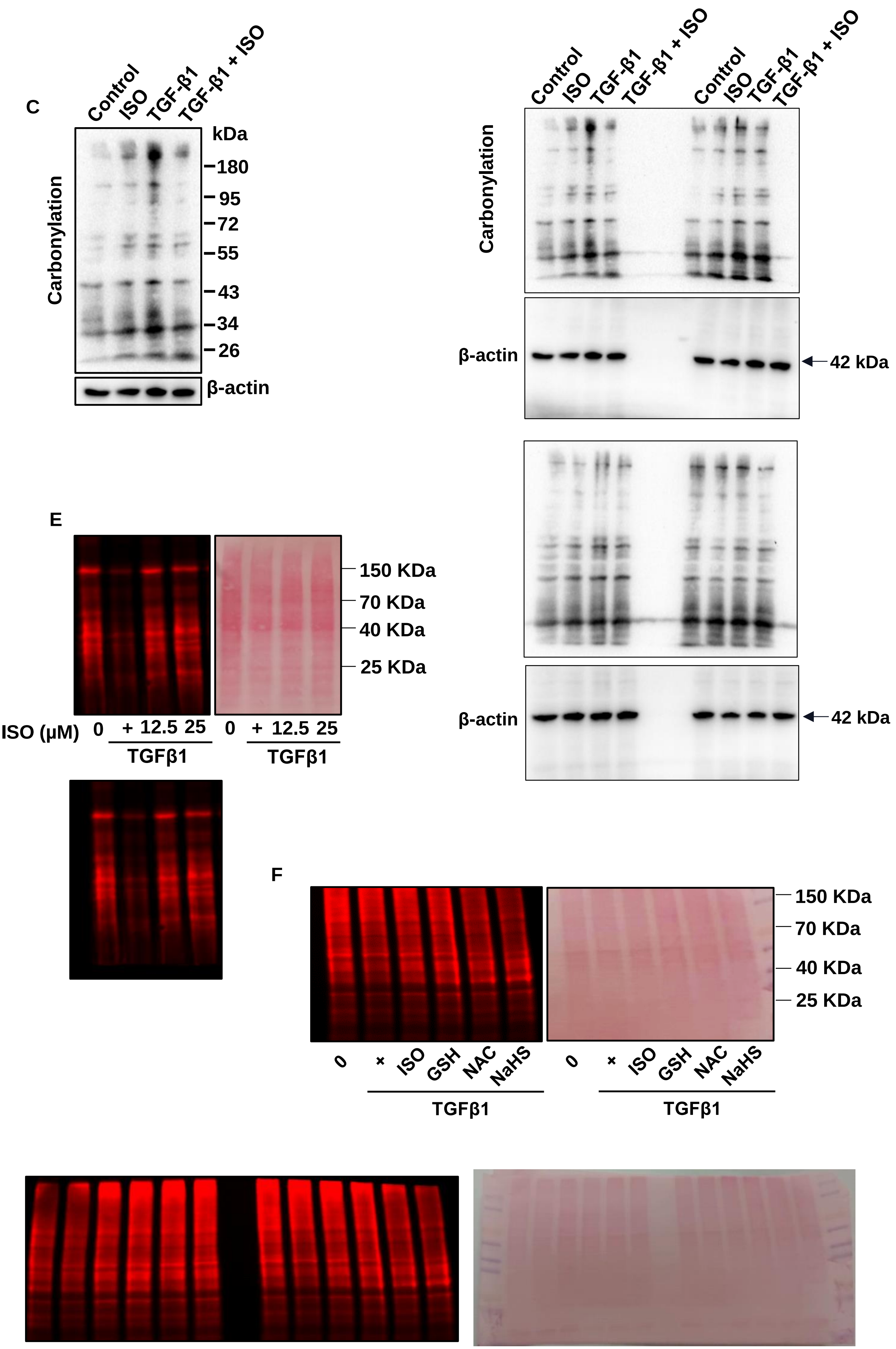

Figure S12: The effect of ISO on Keap1 and Nrf2 interaction in TGF-β1-induced NRK-52E cells

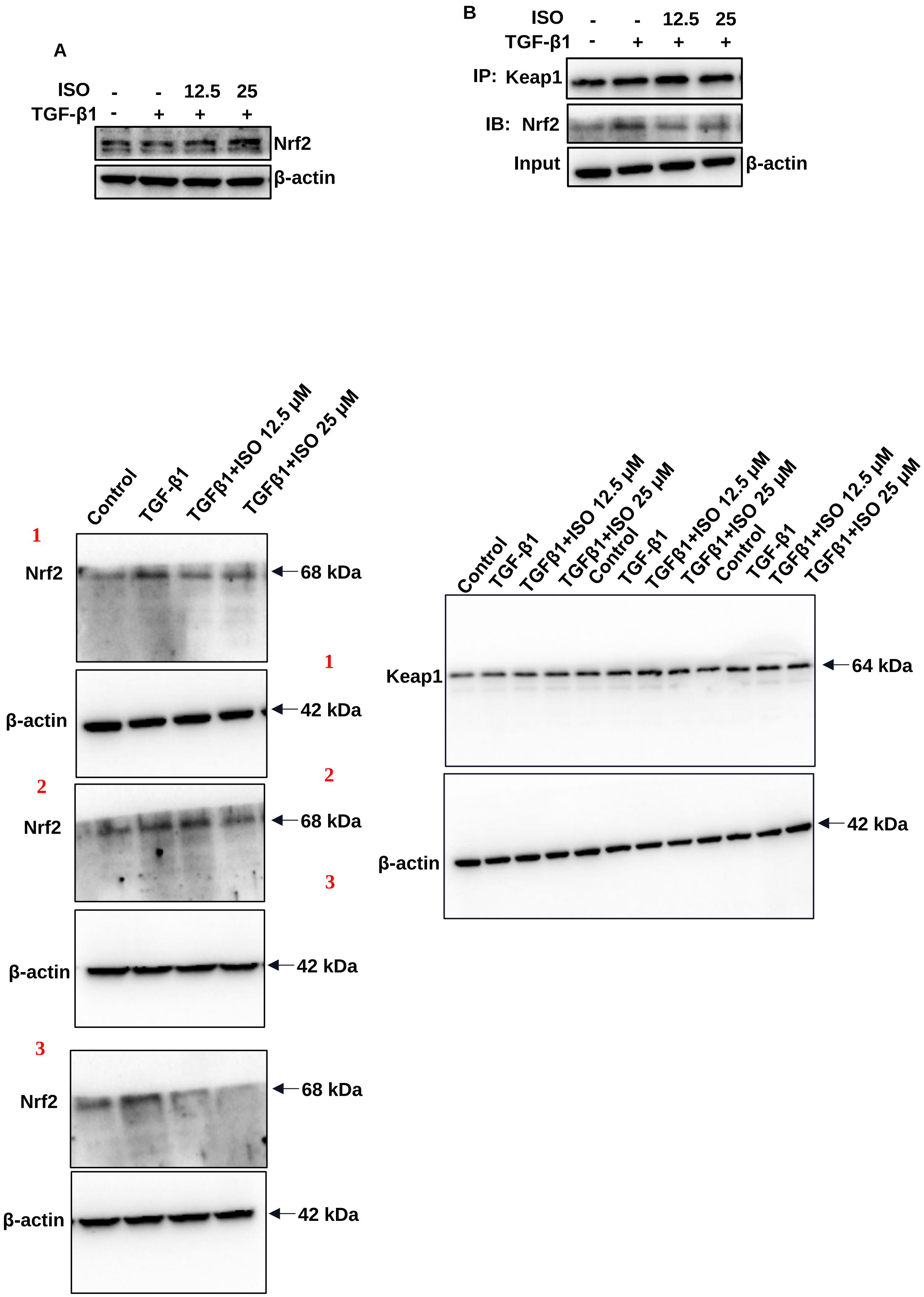

Figure S13: ISO induces Keap1 sulfhydration in TGF-β1-induced NRK-52E cells

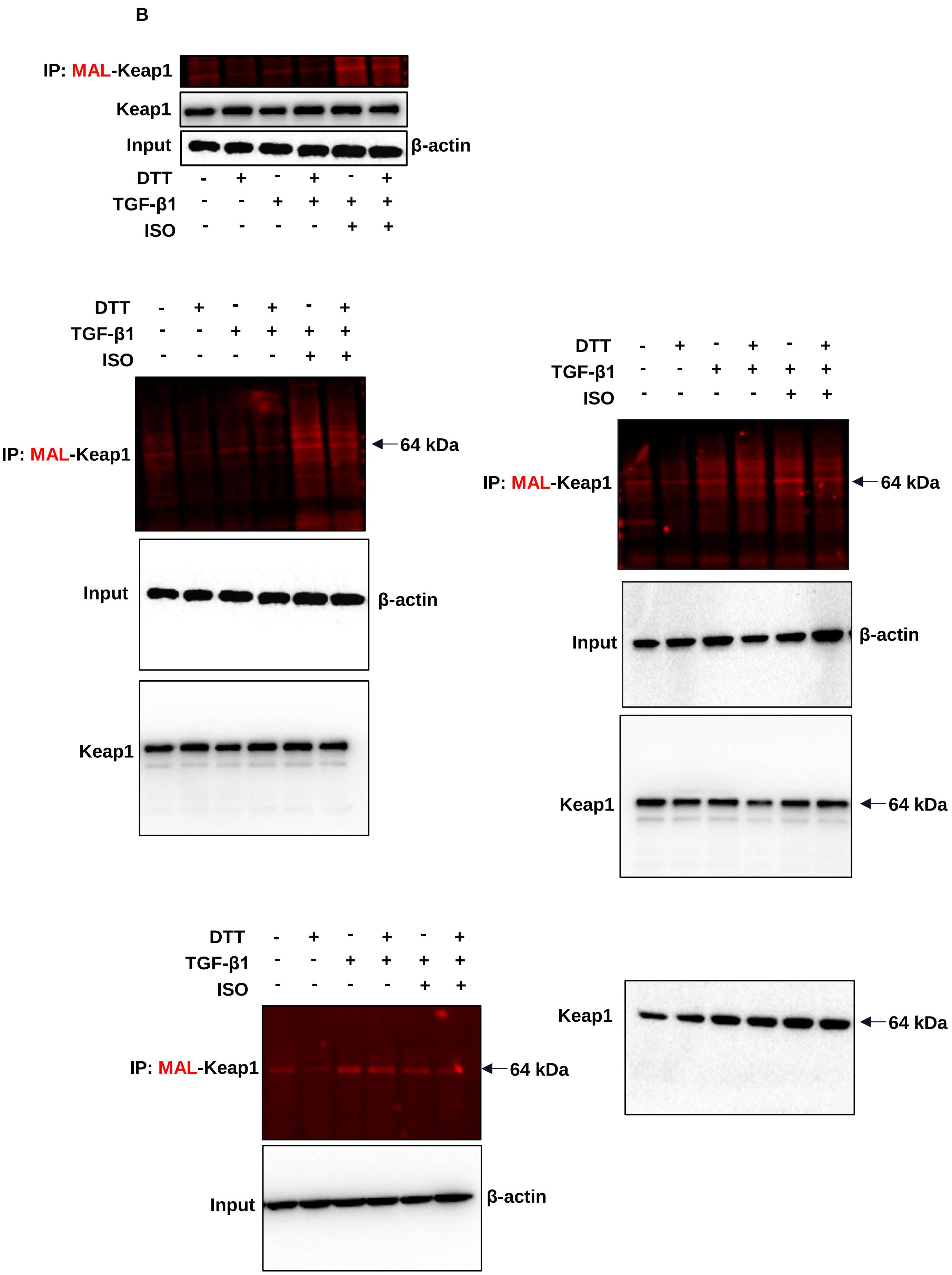

Supplement: Supplementary file 1 [file biomolecules-14-01233-s001.zip › biomolecules-3177629-supplementary.pdf]
